# A New Large-Bodied Oviraptorosaurian Theropod Dinosaur from the Latest Cretaceous of Western North America

**DOI:** 10.1371/journal.pone.0092022

**Published:** 2014-03-19

**Authors:** Matthew C. Lamanna, Hans-Dieter Sues, Emma R. Schachner, Tyler R. Lyson

**Affiliations:** 1 Section of Vertebrate Paleontology, Carnegie Museum of Natural History, Pittsburgh, Pennsylvania, United States of America; 2 Department of Paleobiology, National Museum of Natural History, Smithsonian Institution, Washington, District of Columbia, United States of America; 3 Department of Biology, University of Utah, Salt Lake City, Utah, United States of America; 4 Department of Vertebrate Zoology, National Museum of Natural History, Smithsonian Institution, Washington, District of Columbia, United States of America; Royal Ontario Museum, Canada

## Abstract

The oviraptorosaurian theropod dinosaur clade Caenagnathidae has long been enigmatic due to the incomplete nature of nearly all described fossils. Here we describe *Anzu wyliei* gen. et sp. nov., a new taxon of large-bodied caenagnathid based primarily on three well-preserved partial skeletons. The specimens were recovered from the uppermost Cretaceous (upper Maastrichtian) Hell Creek Formation of North and South Dakota, and are therefore among the stratigraphically youngest known oviraptorosaurian remains. Collectively, the fossils include elements from most regions of the skeleton, providing a wealth of information on the osteology and evolutionary relationships of Caenagnathidae. Phylogenetic analysis reaffirms caenagnathid monophyly, and indicates that *Anzu* is most closely related to *Caenagnathus collinsi*, a taxon that is definitively known only from a mandible from the Campanian Dinosaur Park Formation of Alberta. The problematic oviraptorosaurs *Microvenator* and *Gigantoraptor* are recovered as basal caenagnathids, as has previously been suggested. *Anzu* and other caenagnathids may have favored well-watered floodplain settings over channel margins, and were probably ecological generalists that fed upon vegetation, small animals, and perhaps eggs.

## Introduction

Oviraptorosauria is a clade of maniraptoran theropod dinosaurs with peculiar craniomandibular specializations [1]. Spectacular fossil discoveries have provided abundant information on the morphology, diversity, evolution, and paleobiology of these unusual animals. Oviraptorosaurs ranged in age from at least the first half of the Cretaceous to the very end of this interval, and in body mass from chicken- or turkey-sized taxa [2], [3] to giants hypothesized to weigh in excess of one metric ton [4]. Some Early Cretaceous forms retained teeth [2], [5]–[7], but, by the Late Cretaceous, all known oviraptorosaurs were edentulous. Most if not all oviraptorosaurs were feathered, as evidenced by direct preservation [2], [8], [9], possible quill knobs on the ulna [10], or pygostyle-like terminal caudal vertebrae [11]–[14]. Oviraptorosaurs brooded their nests [15]–[20], employed a reproductive strategy intermediate between those of crocodylians and birds [21], had bird-like brains [22], [23] (but see [24]), and were probably omnivorous or herbivorous [5], [7], [25], [26]. Whereas nearly all recent analyses have interpreted oviraptorosaurs as non-avian maniraptorans, a few others have postulated these theropods as basal birds [27]–[29] or as the sister taxon of Scansoriopterygidae [30], [31], a clade of unusual Jurassic maniraptorans that are frequently placed as basal avians [32], [33].

Most discoveries of oviraptorosaurs have been made in Asia, principally Mongolia and China, and as a result, the balance of our knowledge of the group is derived from fossils found on that continent. Most Late Cretaceous oviraptorosaurs from Asia comprise a clade, Oviraptoridae, which may have been endemic to that landmass. Nevertheless, theropods now recognized as oviraptorosaurs have long been known from North America as well [34]–[38]. Unfortunately, however, nearly all described North American oviraptorosaurian fossils are very incomplete (see Table S1 in File S1), hindering attempts to decipher their anatomy, taxonomy, and phylogenetic affinities. Most authors [1], [39]–[42] have considered all Late Cretaceous North American oviraptorosaurs to be part of a monophyletic assemblage, Caenagnathidae, though other recent studies [31], [43], [44] have argued that taxa traditionally regarded as caenagnathids form a paraphyletic grouping, with some taxa (e.g., *Chirostenotes*, *Hagryphus*) being more closely related to Oviraptoridae than are others (e.g., *Caenagnathus*). Furthermore, despite the fragmentary nature of North American oviraptorosaurian material, many authors [38], [39], [42], [45]–[49] have speculated on the paleoecology of these dinosaurs, often arriving at widely disparate conclusions.

**Figure 1:**
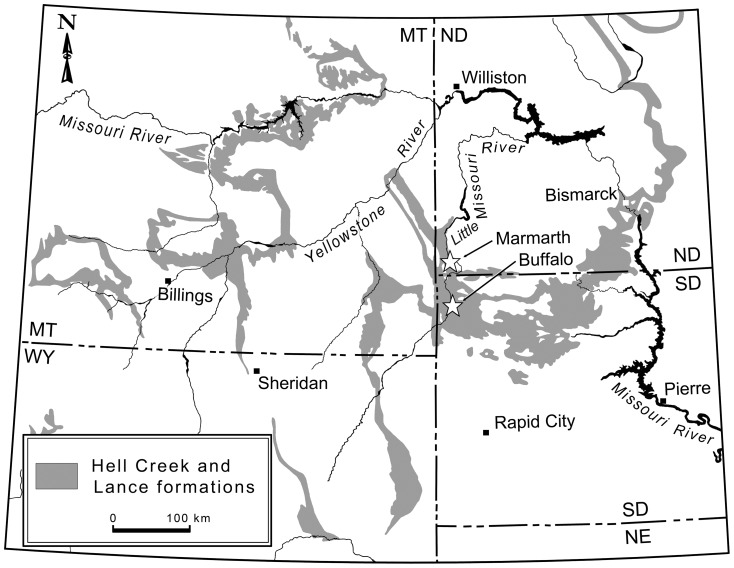
Exposures of the Upper Cretaceous Hell Creek and Lance formations in western North America. Localities that have yielded specimens of *Anzu wyliei* gen. et sp. nov. are marked by white stars. Map modified from [55]. Scale bar  = 100 km.

Here we describe a new, large-bodied (total length ∼3.5 m) oviraptorosaurian taxon based primarily on three well-preserved partial skeletons from the late Maastrichtian of North and South Dakota (Figure 1). Although all of these specimens have been briefly mentioned in the literature [50]–[52], they have never been described in detail. The new taxon offers the first comprehensive picture of the skeletal structure of Caenagnathidae and sheds light on long-standing controversies regarding the taxonomy and interrelationships of North American oviraptorosaurs. Furthermore, its analysis confirms caenagnathid monophyly and provides additional insight into the paleobiology of this enigmatic clade.

## Materials and Methods

### Paleontological ethics statements

Two of the specimens described in this paper (CM 78000, CM 78001) are permanently reposited in the collections of the Section of Vertebrate Paleontology at Carnegie Museum of Natural History, 4400 Forbes Avenue, Pittsburgh, Pennsylvania, United States of America. The third specimen (MRF 319) is permanently reposited in the collections of the Marmarth Research Foundation, 402 South Main Street, Marmarth, North Dakota, United States of America. The latter organization was formally certified under Section 501(c)(3) of the United States Internal Revenue Code for the explicit purposes of research and curation of fossils from exposures of the Hell Creek Formation in southwestern North Dakota and neighboring regions. The fossils currently in the trust of the collection are intended for the establishment of a museum in the town of Marmarth, and are presently housed and curated in a dedicated facility in that town. Qualified researchers who wish to access this collection should direct such requests to the fourth author (T.R.L.) or to Ms. Barbara Benty (bbentysac@sbcglobal.net). Complete sets of casts of MRF 319 will also be deposited in the collections of the Section of Vertebrate Paleontology at Carnegie Museum of Natural History and the Department of Paleobiology at the National Museum of Natural History, 1000 Constitution Avenue NW, Washington, District of Columbia, United States of America.

Detailed locality information for the CM specimens is on file in the Section of Vertebrate Paleontology at Carnegie Museum of Natural History and is available to qualified researchers upon request; that for MRF 319 is on file at the Marmarth Research Foundation and is available to qualified researchers upon request. No permits were required for the described study, which complied with all relevant regulations. All specimens were collected from privately-owned land in the United States of America with the written consent of the respective landowners.

### Institutional abbreviations

BHM, Black Hills Institute of Geological Research, Hill City, South Dakota, United States of America; CM, Carnegie Museum of Natural History, Pittsburgh, Pennsylvania, United States of America; CMN, Canadian Museum of Nature, Ottawa, Ontario, Canada; FMNH, Field Museum of Natural History, Chicago, Illinois, United States of America; MOR, Museum of the Rockies, Bozeman, Montana, United States of America; MRF, Marmarth Research Foundation, Marmarth, North Dakota, United States of America; TMP, Royal Tyrrell Museum of Palaeontology, Drumheller, Alberta, Canada.

### Nomenclatural acts

The electronic edition of this article conforms to the requirements of the amended International Code of Zoological Nomenclature, and hence the new names contained herein are available under that Code from the electronic edition of this article. This published work and the nomenclatural acts it contains have been registered in ZooBank, the online registration system for the ICZN. The ZooBank LSIDs (Life Science Identifiers) can be resolved and the associated information viewed through any standard web browser by appending the LSID to the prefix “http://zoobank.org/.” The LSID for this publication is: urn:lsid:zoobank.org:pub: 41FF30C8-8E27-45A5-A250-13937D6206F3. The electronic edition of this work was published in a journal with an ISSN, and has been archived and is available from the following digital repositories: PubMed Central and LOCKSS.

## Results

### Systematic paleontology

Theropoda Marsh 1881 [53]

Oviraptorosauria Barsbold 1976 [54]

Caenagnathidae Sternberg 1940 [37]

*Anzu* gen. nov.

urn:lsid:zoobank.org:act:B4E094D2-8081-452E-8976-DB96DA97308D

Anzu wyliei sp. nov.

urn:lsid:zoobank.org:act:E164FB53-289F-4B0E-916C-C581F2DE57A4

#### Holotype

**Figure 2:**
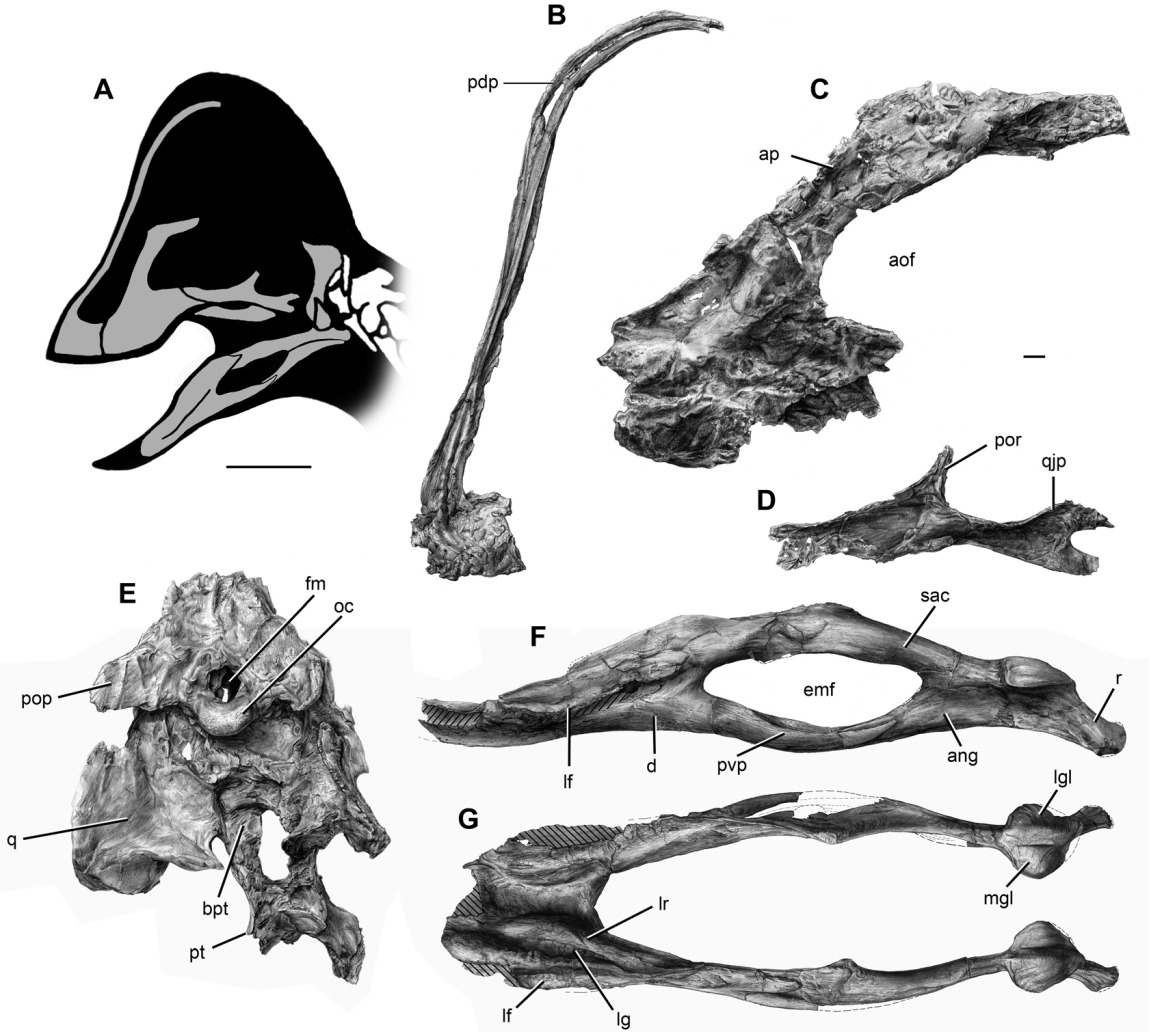
Craniomandibular skeleton of *Anzu wyliei* gen. et sp. nov. (**A**) Reconstructed skull and mandible in left lateral view, with preserved bones in gray. (**B**) Left premaxilla of CM 78001 in lateral view. (**C**) Left maxilla of CM 78001 in lateral view. (**D**) Left jugal of CM 78001 in lateral view. (**E**) Braincase with articulated quadrates and pterygoids of CM 78001 in posterior view. Reconstructed mandible of CM 78000 in left lateral (**F**) and dorsal (**G**) views (hatching indicates broken areas, dashed lines indicate restoration). Abbreviations: ang, angular; aof, antorbital fenestra; ap, ascending process; bpt, basipterygoid process; d, dentary; emf, external mandibular fenestra; fm, foramen magnum; lf, lateral flange; lg, lateral groove; lgl, lateral facet of mandibular glenoid; lr, lingual ridge; mgl, medial facet of mandibular glenoid; oc, occipital condyle; pdp, posterodorsal process; pop, paroccipital process; por, postorbital process; pt, pterygoid; pvp, posteroventral process; q, quadrate; qjp, quadratojugal process; r, retroarticular process; sac, surangular–articular–coronoid complex. Scale bars  = 10 cm in A; 1 cm in B–G.

**Figure 3:**
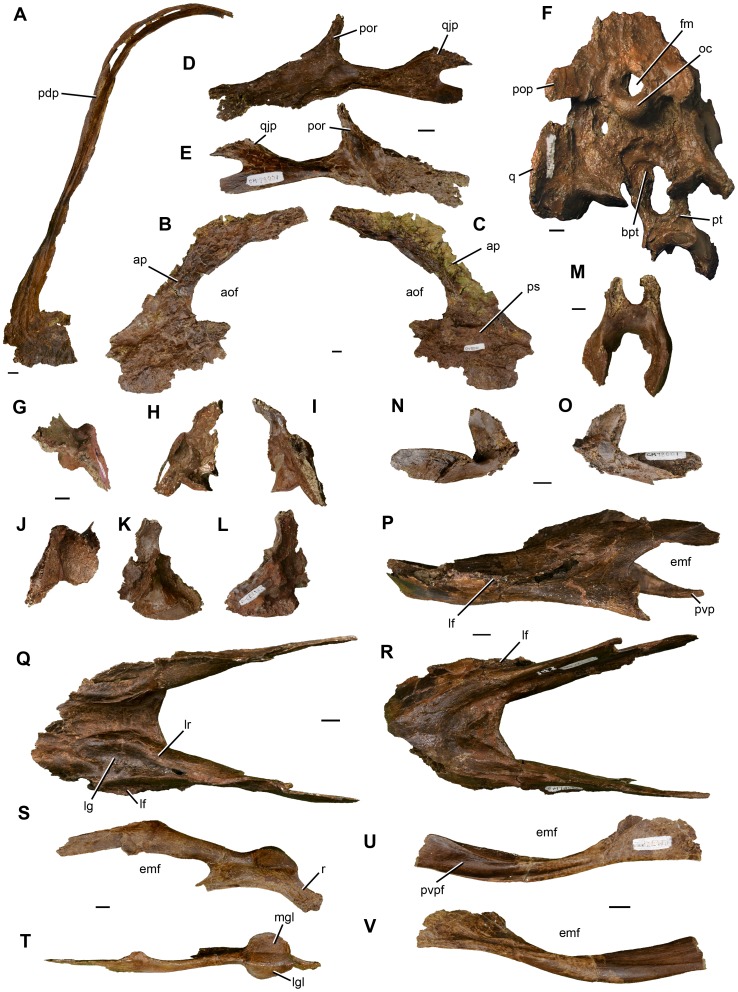
Photographs of craniomandibular elements of *Anzu wyliei* gen. et sp. nov. (**A**) Left premaxilla of CM 78001 in lateral view. Left maxilla of CM 78001 in lateral (**B**) and medial (**C**) views. Left jugal of CM 78001 in lateral (**D**) and medial (**E**) views. (**F**) Braincase with articulated quadrates and pterygoids of CM 78001 in posterior view. Right quadrate of CM 78000 in dorsal (**G**), anterior (**H**), posterior (**I**), ventral (**J**), medial (**K**), and lateral (**L**) views. (**M**) Fused pterygoids of CM 78000 in ventral view. Right ectopterygoid of CM 78001 in lateral (**N**) and medial (**O**) views. Fused dentaries of CM 78000 in left lateral (**P**), dorsal (**Q**), and ventral (**R**) views. Left surangular–articular–coronoid complex of CM 78000 in lateral (**S**) and dorsal (**T**) views. Left angular of CM 78000 in lateral (**U**) and medial (**V**) views. Abbreviations: aof, antorbital fenestra; ap, ascending process; bpt, basipterygoid process; emf, external mandibular fenestra; fm, foramen magnum; lf, lateral flange; lg, lateral groove; lgl, lateral facet of mandibular glenoid; lr, lingual ridge; mgl, medial facet of mandibular glenoid; oc, occipital condyle; pdp, posterodorsal process; pop, paroccipital process; por, postorbital process; ps, palatal shelf; pt, pterygoid; pvp, posteroventral process; pvpf, facet for posteroventral process of dentary; q, quadrate; qjp, quadratojugal process; r, retroarticular process. Scale bars  = 1 cm.

**Figure 4:**
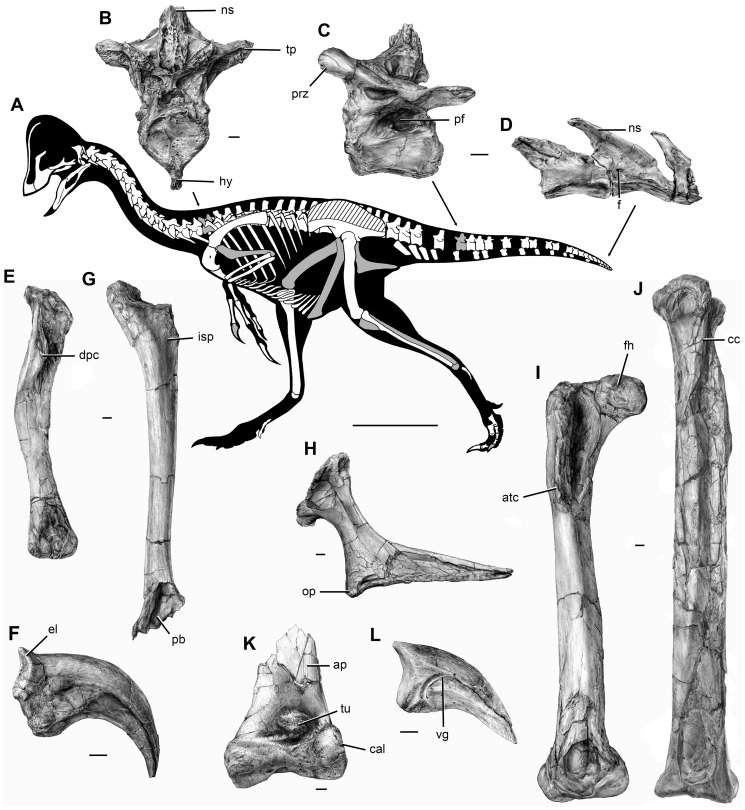
Postcranial skeleton of *Anzu wyliei* gen. et sp. nov. as preserved in the CM specimens. (**A**) Skeletal reconstruction in left lateral view, with illustrated bones in gray and other preserved bones in white (hatching indicates heavily reconstructed portions of the ilia of CM 78001). (**B**) Anterior dorsal vertebra of CM 78001 in anterior view. Anterior (**C**) and posteriormost preserved (**D**) caudal vertebrae of CM 78000 in left lateral view. (**E**) Right humerus of CM 78000 in anterior view. (**F**) Manual ungual I of CM 78000 in lateral view. Left pubis (**G**) and ischium (**H**) of CM 78001 in lateral view. Right femur (**I**) and left tibia (**J**) and astragalocalcaneum (**K**) of CM 78000 in anterior view. (**L**) Pedal ungual of CM 78000 in lateral view. Abbreviations: ap, ascending process; atc, ‘accessory trochanteric crest’; cal, calcaneum; cc, cnemial crest; dpc, deltopectoral crest; el, extensor ‘lip’; f, foramen; fh, femoral head; hy, hypapophysis; isp, ischial peduncle; ns, neural spine; op, obturator process; pb, pubic ‘boot’; pf, pneumatic fossa; prz, prezygapophysis; tp, transverse process; tu, tubercle; vg, vascular groove. Scale bars  = 50 cm in A; 1 cm in B–L.

CM 78000, a disarticulated but closely associated partial skeleton that includes premaxilla fragments, the incomplete braincase, both quadrates and pterygoids, additional cranial fragments, the nearly complete but disarticulated mandible, multiple cervical and caudal vertebrae, numerous cervical and dorsal ribs, gastralia, and hemal arches, both scapulocoracoids, the right humerus, the left radius, ulna, and metacarpal II, several manual phalanges including unguals, both femora, tibiae, fibulae, astragalocalcanea, and first metatarsals, the incomplete left metatarsal V, and several pedal phalanges including unguals (Figures 2, 3, 4).

#### Referred specimens

**Figure 5:**
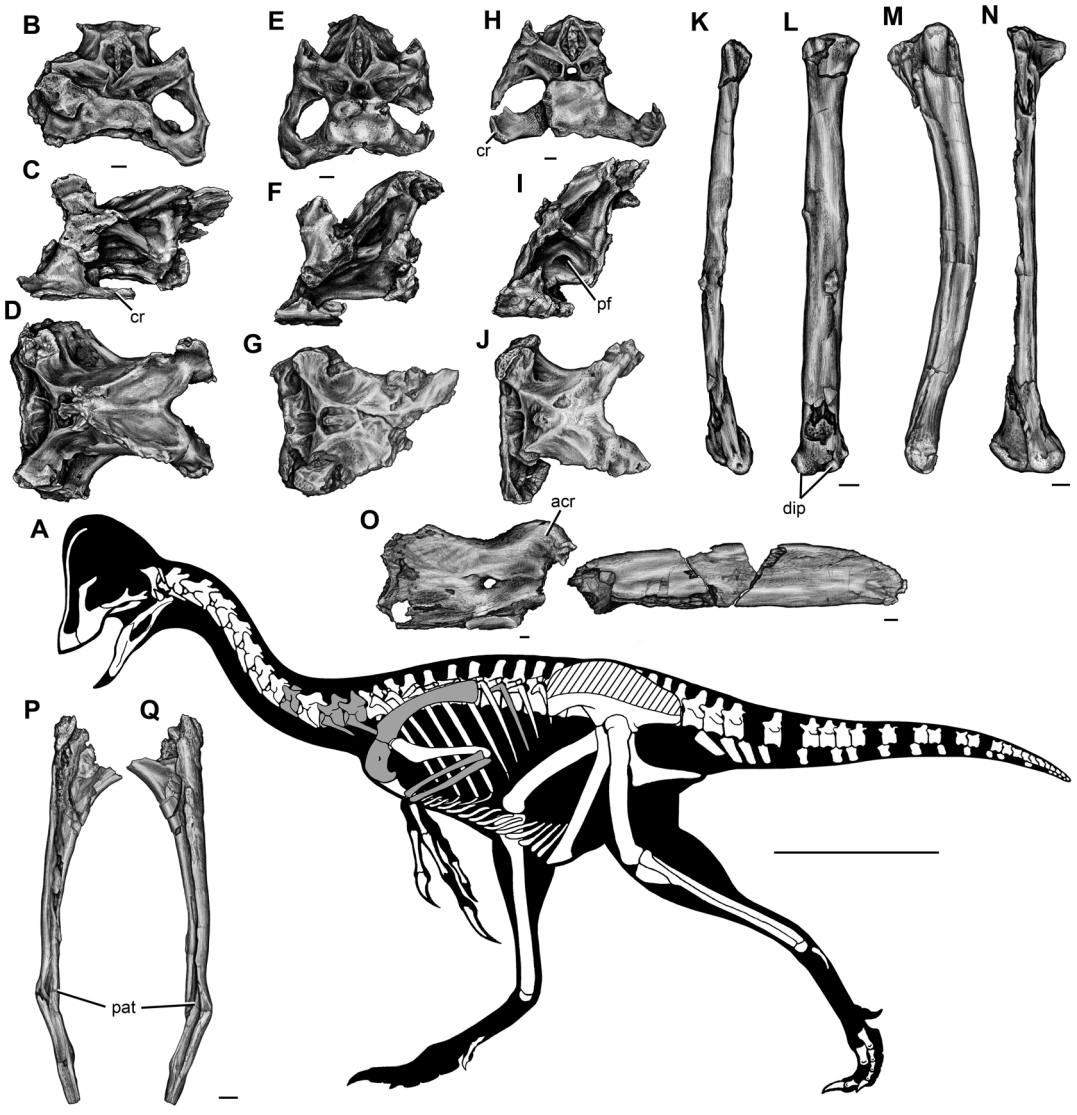
MRF 319, a partial oviraptorosaurian skeleton referred to *Anzu wyliei* gen. et sp. nov. (**A**) Skeletal reconstruction in left lateral view, with preserved bones in gray and bones represented in other *Anzu* specimens in white (hatching indicates heavily reconstructed portions of the ilia of CM 78001). Middle-posterior (ninth?) cervical vertebra in (**B**) anterior, (**C**) left lateral, and (**D**) dorsal views. Posterior (11^th^?) cervical vertebra in (**E**) anterior, (**F**) left lateral, and (**G**) dorsal views. Posterior (12^th^?) cervical vertebra in (**H**) anterior, (**I**) left lateral, and (**J**) dorsal views. Anteroposteriorly crushed left radius in lateral (**K**) and anterior (**L**) views. Mediolaterally crushed left ulna in lateral (**M**) and anterior (**N**) views. (**O**) Partial left scapulocoracoid in lateral view. Dorsal rib in anterior (**P**) and posterior (**Q**) views. Abbreviations: acr, acromial process; cr, cervical rib; dip, distal processes; pat, pathology; pf, pneumatic fossa. Scale bars  = 50 cm in A; 1 cm in B–Q.

CM 78001, a mostly disarticulated but closely associated partial skeleton that includes the nearly complete left and fragmentary right premaxillae, maxillae, and jugals, the articulated braincase, quadrates, and pterygoids, the right ectopterygoid, additional cranial fragments, all cervical and dorsal vertebrae, the incomplete sacrum, several caudal vertebrae, numerous dorsal ribs, gastralia, and hemal arches, both sternal plates, ilia, pubes, ischia, femora, tibiae, fibulae, and astragalocalcanea, the left metatarsal V, and several pedal phalanges including unguals (Figures 2, 3, 4); MRF 319, an associated partial postcranial skeleton that includes three cervical vertebrae, a dorsal rib, and the left scapulocoracoid, radius, and ulna (Figure 5); FMNH PR 2296 (formerly BHM 2033), a coossified left surangular–articular–coronoid complex [46].

#### Etymology

The genus name is for Anzu, a feathered demon in ancient Mesopotamian (Sumerian and Akkadian) mythology, and alludes to the distinctive appearance of this large, presumably feathered dinosaur. The species name is for Mr. Wylie J. Tuttle, grandson of Mr. and Mrs. Lee B. Foster, in recognition of Mr. and Mrs. Foster's generous support of the scientific research and collections activities at Carnegie Museum of Natural History.

#### Localities and horizon

Harding County, South Dakota, United States of America (CM 78000, CM 78001 [skeletons separated by only ∼100 m]; FMNH PR 2296); Slope County, North Dakota, United States of America (MRF 319) (Figure 1). Upper Cretaceous (upper Maastrichtian [55]) Hell Creek Formation.

The holotype of *Anzu wyliei* gen. et sp. nov. (CM 78000) was recovered from an exposure on privately-owned land approximately 23 km southwest of the town of Buffalo, South Dakota (Figure 1). The fossil horizon is a medium- to thick-bedded, greenish-gray silty mudstone with secondary silicification that is interpreted to represent a low-energy, floodplain depositional setting. The skeleton was disarticulated with minor association between neighboring elements. Limited current transport was evident, with bones showing a preferred orientation to the north-northeast and north-northwest. Normal faults with minor (5–40 cm) vertical displacement were commonly found during the excavation (M. Triebold and W. Stein, pers. comm.).

CM 78001 comes from a site approximately 100 m east of the type locality, and from a stratigraphic level of the Hell Creek Formation that is approximately 3.5 m lower in section. The specimen was recovered from a calcite-cemented, medium yellow-brown silty mudstone with abundant organic matter that is interpreted to represent a low-energy, floodplain depositional environment adjacent to a swampy area, as indicated by an overlying coal seam. Elements of CM 78001 were found in close association with some articulation. The specimen was eroding from a low, vegetated knob, and as such, many of its bones exhibit damage from weathering and invasion by plant roots. Like that of CM 78000, the CM 78001 site displayed several normal faults with minor (2–25 cm) vertical displacement. Bones that spanned these fault zones (e.g., the right femur) were broken and separated, with up to 25 cm of vertical and 30 cm of horizontal displacement, respectively (M. Triebold and W. Stein, pers. comm.).

A large concentration of organic debris and small fossils (e.g., mollusk shells, bones of small vertebrates) was recovered between the dorsal ribs and pelvis of CM 78001. This debris was much more prevalent within this region than elsewhere in the quarry (M. Triebold and W. Stein, pers. comm.). Consequently, there is some possibility that it may represent gut contents; nevertheless, because CM 78001 was mostly disarticulated, this is difficult to demonstrate with certainty. Contamination from fluvial processes is a likely alternative explanation for the presence of this material.

MRF 319 was collected from the upper one-third of the Hell Creek Formation, from an exposure on privately-owned land approximately 5 km northwest of the town of Marmarth, North Dakota. It was found in a poorly consolidated sandstone rich in clay rip-up clasts that is herein interpreted as a channel lag deposit. The specimen was associated with numerous gar scales and a single water-worn bone of an indeterminate ornithischian dinosaur.

#### Diagnosis

Caenagnathid oviraptorosaur diagnosed by the following autapomorphies (characters observable in multiple specimens are denoted with an asterisk; hypothesized reversals are denoted with a dagger): (1) tall, crescentic cranial crest formed by posterodorsal processes of premaxillae*; (2) body of maxilla lacking antorbital fossa; (3) maxillary ascending process elongate and shaped like an inverted ‘L’; (4) quadratojugal process of jugal dorsoventrally deep^†^; (5) quadratojugal process of jugal bifurcated posteriorly^†^; (6) occipital condyle transversely wider than foramen magnum*^†^; (7) prominent lateral flange on symphyseal region of dentary; (8) elongate retroarticular process of mandible (retroarticular process subequal in anteroposterior length to quadrate articulation)*; (9) distal end of radius divided into two rounded processes*; (10) sulcus on ventromedial aspect of manual phalanx II-1; (11) tubercle on anterior surface of astragalus near base of ascending process*. Differs from hypothesized sister taxon *Caenagnathus collinsi* in possessing the following mandibular features, in addition to autapomorphies (7) and (8): (1) considerably larger size*; (2) anterodorsal margin of dentary only shallowly concave in lateral view; (3) four or five, rather than six, ‘lateral occlusal grooves’ on dentary; (4) posterodorsal process of dentary not extending to anteroposterior level of coronoid process*; (5) anteroventral flange of surangular short (such that dentary forms anterodorsal border of external mandibular fenestra)*; (6) external mandibular fenestra much greater in dorsoventral diameter (due to ventral bowing of angular and posteroventral process of dentary); (7) medial and lateral facets of mandibular glenoid subequal in width*. Differs from comparably-sized but stratigraphically older caenagnathid *Hagryphus giganteus* in possessing autapomorphy (10) and in having more slender proximal phalanges of manual digits I and II (mediolateral shaft width approximately 10% of maximum proximodistal length versus approximately 15% in *H*. *giganteus*).

#### Taxonomic comments

We assign these four oviraptorosaur specimens to *Anzu wyliei* gen. et sp. nov. on the following grounds. Autapomorphies (1) and (6)–(11) are all observable in the holotype of *A*. *wyliei*, CM 78000. Each of the three referred specimens (CM 78001, MRF 319, and FMNH PR 2296) shares at least one of these autapomorphies with CM 78000, justifying referral to *A*. *wyliei*. Specifically, CM 78001 shares autapomorphies (1), (6), and (11), MRF 319 shares autapomorphy (9), and FMNH PR 2296 shares autapomorphy (8). Furthermore, the referral of CM 78001 to *A*. *wyliei* broadens the diagnosis of the new taxon to include autapomorphies (2)–(5), which are, at present, observable only in that specimen.

### Description and comparisons

#### Craniomandibular skeleton

The most prominent cranial feature of *Anzu wyliei* is a very tall, crescentic median crest formed by the greatly elongated posterodorsal processes of the premaxillae (Figures 2A, 2B, 3A). Although cranial crests are present in many oviraptorids, only that of *Rinchenia* [1], [56] bears any resemblance to the crest of the new taxon. The ventral margin of the premaxilla is edentulous and crenulated as in other oviraptorosaurs [1], though the crenulations are less pronounced than in some oviraptorids. The maxilla (Figures 2C, 3B, 3C) has a well-developed ascending process that first extends posterodorsally before turning posteriorly, and that is anteroposteriorly longer than those of oviraptorids. The maxilla is dorsoventrally deeper ventral to the antorbital fenestra than that of *Epichirostenotes* [40], [57]. There is no indication of the distinct medial inset of the maxillary body that occurs in oviraptorids [1]. The jugal (Figures 2D, 3D, 3E) appears remarkably plesiomorphic for an oviraptorosaur: both the maxillary and quadratojugal processes are dorsoventrally deep, and the latter is divided into two lobe-shaped projections posteriorly. This is similar to the condition in non-oviraptorosaurian theropods but unlike all other oviraptorosaurs for which this part of the jugal is known, including basal forms (e.g., *Caudipteryx* [2], [8], *Avimimus* [58]). The postorbital process of the jugal is posterodorsally angled, as in most uncrested oviraptorosaurs [1] and *Nemegtomaia* [20], [59]. Unlike the condition in other oviraptorosaurs, the occipital condyle is transversely wider than the foramen magnum (Figures 2E, 3F), a character that may be related to the large size of *Anzu* compared to almost all other members of this clade (P. Currie, pers. comm.). The paroccipital processes extend ventrolaterally, but are shorter and more laterally oriented than in *Epichirostenotes* [40]. In contrast to that of at least some oviraptorids, the basisphenoid has short but distinct basipterygoid processes. The quadrate of *Anzu* (Figures 2E, 3F–L) appears to be pneumatized, as in oviraptorids, but its ventrolateral extreme lacks the distinctive accessory process for articulation with the quadratojugal that is present in members of that clade [1]. At its approximate dorsoventral midpoint, the posterior end of the lateral surface does bear a low, angular projection for contact with the quadratojugal. This projection forms the anterodorsal margin of a shallow cotyle. The mandibular articulation is deeply divided into slightly convex lateral and medial condyles by an anteromedially extending groove. The condyles are subequal in width and inclined relative to each other at a nearly right angle. The pterygoid flange of the quadrate extends anteromedially and has a deeply recessed medial area for contact with the pterygoid. The pterygoids are fused medially, forming a robust, X-shaped structure (Figures 2E, 3F, 3M). The ectopterygoid (Figures 3N, 3O) differs from that of oviraptorids in being anteroposteriorly short and in having a hook-like jugal process.

The mandibular symphysis of *Anzu* is subhorizontal in lateral view, transversely broad, and extensively pneumatized (Figures 2F, 2G, 3P–R). It is firmly fused without an interdentary suture, as in other definitive caenagnathids and *Gigantoraptor* but unlike basal oviraptorosaurs (with the possible exception of *Incisivosaurus* [6]), *Microvenator* [60], and oviraptorids. The anteroposteriorly elongate, dorsoventrally shallow symphysis more closely resembles that of *Caenagnathus collinsi* [37] than it does that of ‘*Caenagnathus*’ *sternbergi* [41], [46]. It differs markedly from the short, deep symphysis of almost all other oviraptorosaurs, including *Caenagnathasia* [46], *Caudipteryx* [8], *Gigantoraptor* [4], *Leptorhynchos* [42], *Microvenator* [60], and oviraptorids [1]. The dentary (Figures 2F, 2G, 3P–R) has a prominent lateral flange that spans much of its length, becoming especially pronounced lateral to the symphyseal region. A comparable lateral flange is present in *Gigantoraptor* [4], but it is restricted to the region posterior to the symphysis in that taxon. Like the premaxilla and maxilla, the dentary lacks teeth, but as in other derived caenagnathids [42], [46], a deep, longitudinal groove extends across its dorsal surface, close to the lateral edge. As in other caenagnathids, this groove is bounded laterally by a tall ridge, the medial surface of which is incised by a series of four or five ‘lateral occlusal grooves’ (*sensu* Currie et al. [46]). The dentary bifurcates posteriorly to frame the anterior margin of the large, anteroposteriorly elongate external mandibular fenestra. The posteroventral process is noticeably bowed ventrally, as in *Gigantoraptor* [4] and mandibles referred to ‘*C*.’ *sternbergi* [41], [42], [46]. Unlike in oviraptorids, no anterior process of the surangular projects into the external mandibular fenestra. The surangular has a low coronoid eminence and is coossified with the articular and coronoid (Figures 2F, 2G, 3S, 3T). It lacks foramina, as in all other oviraptorosaurs in which this condition can be evaluated except *Banji* [61] and some individuals of *Yulong* [3]. The posterior end of the mandibular ramus differs from that of ‘*C*.’ *sternbergi* [38], [41] in having a proportionally shallower surangular, mandibular glenoid, and retroarticular process. Nevertheless, the glenoid is distinctly convex and elevated in lateral view, as in all other oviraptorosaurs except *Avimimus* [58]. In contrast to the condition in most oviraptorids, the angular (Figures 2F, 2G, 3U, 3V) contributes extensively to the ventral border of the external mandibular fenestra.

#### Postcranial axial skeleton

The complete presacral vertebral series of CM 78001 (Figures 4A, 4B) consists of 22 vertebrae (12 cervicals and ten dorsals). The cervical ribs of *Anzu* are fused to their respective vertebrae (Figures 5B–J), as in *Avimimus* [1] and the oviraptorid *Heyuannia* [62]. The centra of the posteriormost cervical and all dorsal vertebrae have lateral pneumatic fossae (‘pleurocoels’). Anterior dorsal centra (Figure 4B) bear prominent hypapophyses that are rounded in lateral view. The sacrum comprises six ankylosed vertebrae, as in some oviraptorosaurs (e.g., *Chirostenotes pergracilis* [39], *Epichirostenotes* [40]) but not others (e.g., derived oviraptorids, which have seven or eight sacrals [20], [59], [62]). The centra of the sacral and all but the most posterior caudal vertebrae have lateral pneumatic fossae (Figure 4C). As in *Caudipteryx* [8], the caudal series terminates in a pygostyle-like conformation consisting of highly modified but unfused vertebrae (Figure 4D). Comparable conditions also occur in *Similicaudipteryx* [13], *Nomingia* [11], [12], [14], and the oviraptorids *Citipati* and *Conchoraptor* [14], but the terminal vertebrae are coossified in these taxa. Consequently, pygostyle-like structures appear to be widespread among Oviraptorosauria.

#### Appendicular skeleton

The appendicular skeleton of *Anzu* is well represented. The sternal plates resemble those of oviraptorids but differ from *Caudipteryx* in having well-developed, paired lateral processes, the more anterior of which is confluent with the anterior margin of the bone. The posterolateral region of each sternal plate is pierced by a foramen. The scapula and coracoid are firmly fused (Figure 5O). The anteroposteriorly short acromial process is laterally everted and projects anteroventrally at an obtuse angle to the long axis of the scapular blade. The anteroposteriorly short coracoid has an elongate, pointed posteroventral process. The humerus (Figure 4E) is bowed laterally as in *Gigantoraptor* [4] and strongly flexed posteriorly at midshaft, such that, when seen in lateral view, its anterior margin is concave and its posterior margin is convex. The anterodistal surface of the deltopectoral crest exhibits a lateral muscle scar that marks an origin of *M. biceps brachii*. The deltopectoral crest is low and strongly medially deflected, as in *Gigantoraptor*, differing from that of oviraptorids (e.g., *Ingenia* [1]), which tends to be better developed and restricted to the lateral margin of the bone. The radius (Figures 5K, 5L) is bowed anteriorly, and its distal end is divided into two rounded processes. The ulna (Figures 5M, 5N) is gently bowed posteriorly. Metacarpal II is elongate, being 40.3% the length of the humerus (see Table S2 in File S1). The ventromedial surface of manual phalanx II-1 is marked by a clearly defined, proximoventrally–distodorsally oriented groove. The proximodorsal margin of the articular cotyle of each manual ungual is attenuated into an unusually prominent extensor ‘lip,’ as in *C*. *pergracilis* [34], [39], *Elmisaurus rarus* [63], and *Hagryphus* [64] (Figure 4F). The ilia of CM 78001 are poorly preserved, but the preacetabular process appears to have a deeply concave ventral margin that extends ventrally below the dorsal margin of the acetabulum. The pubic peduncle is anteroposteriorly longer than the ischial peduncle. The pubis (Figure 4G) is straight-shafted, unlike the pubes of oviraptorids [1], and approximately one-third longer than the ischium. The medial surface of its ischial peduncle is excavated by a well-defined, posteriorly circumscribed fossa, as in some other caenagnathids (i.e., *Epichirostenotes*, *Ojoraptorsaurus* [57]). The pubes meet medially in an anteroposteriorly thin, proximodistally elongate ‘apron’ that terminates proximal to the distal ‘boot.’ The pubic ‘boot’ projects further anteriorly than posteriorly, and its distal surface is distinctly concave, especially posteriorly. When the pelvis is articulated, the pubis projects anteroventrally, as in most other non-avian theropods. The ischium (Figure 4H) is almost identical to those of *C. pergracilis* [39] and *Epichirostenotes* [40], and also closely resembles that of *Nomingia* [12]. The wide, plate-like, laterally concave ischial shaft is strongly curved posterodistally. The anteriorly concave obturator process is positioned near the proximodistal midline of the shaft, and the obturator foramen is reduced to a notch. The apex of the obturator process is not angled distally as in some derived oviraptorids (e.g., *Heyuannia* [62]). The femur (Figure 4I) is approximately 1.5 times longer than the humerus. The long axis of the femoral head and neck meets that of the shaft at an approximate right angle. The greater and anterior (lesser) trochanters are fused into a trochanteric crest, which is separated from the head by a shallow proximal embayment. Further distally, a distinct ‘accessory trochanteric crest’ arises from the anterolateral edge of the shaft, as in *C. pergracilis* [39] and *Microvenator* [60]. There is no fourth trochanter. The lateral (fibular) condyle of the femur projects further distally than the medial, though this condition is not nearly as pronounced as it is in some oviraptorids (e.g., *Ingenia* [65]). The femur of CM 78000 is slightly less robust than that of CM 78001 (see Table S2 in File S1). The tibia (Figure 4J) is gracile and 1.25 times longer than the femur. The medial surface of the proximal end of the fibula is excavated by a deep fossa. The astragalus is fused to the calcaneum (Figure 4K) but not to the tibia. A shallow, mediolaterally oriented sulcus demarcates the tall, subtriangular ascending process from the remainder of the astragalus. Distally, the anterior surface of this process is marked by a rugose, subcircular tubercle that is placed slightly lateral to the mediolateral midline of the element and that has not been described in other oviraptorosaurs. Several oviraptorids have a well-defined depression in this area [1], whereas the recently described oviraptorosaur *Nankangia* has a pair of proximodistally elongate, subparallel fossae [66]. Most of the metatarsus is unknown in *Anzu*. The pedal unguals (Figure 4L) have paired lateral vascular grooves, as in *Avimimus* [67], *Gigantoraptor* [4], and several other oviraptorosaurian specimens (e.g., MOR 752 [47]); these are separate proximally but become confluent near the proximodistal midpoint of each claw.

#### Pathologies

Pathological bones have been identified in two individuals of *Anzu*: a proximal pedal phalanx (possibly phalanx IV-1) of CM 78000 and the dorsal rib of MRF 319. Because pathological features may provide insights into the paleobiology of extinct taxa [68], we briefly comment on these here. The phalanx exhibits a large bony exostosis on the dorsal aspect of its distal articular surface. There are erosional pits on this articular surface, probably representing subchondral bone cysts resulting from arthritic changes. This pathology likely arose from a regional trauma (e.g., a tendon avulsion) rather than an infection, due to the limited scope of the damage (D. Malleske, pers. comm.); however, a histological analysis is required for a more definitive diagnosis. The dorsal rib of MRF 319 bears an enlarged, asymmetrical callus near the distal end of the element (Figures 5P, 5Q), indicating that this individual may have experienced a broken and healed rib.

### Phylogenetic analysis

We conducted a phylogenetic analysis to investigate the affinities of *Anzu wyliei* within Oviraptorosauria. The analysis was modified from that recently published by Longrich et al. [42], which was, in turn, based on previous studies [1], [69]. We modified the character-taxon matrix of Longrich et al. [42] in multiple ways, as detailed below.

#### Characters

We altered the descriptions of eight characters analyzed by Longrich et al. [42], either following another recent phylogenetic analysis of Oviraptorosauria [3] (characters 1, 3, 9, 134, and 144) or in novel ways (characters 92, 158, and 177). We also added a total of 25 characters (our characters 206–230): 14 of these were drawn from other previous analyses [3], [61], [70], [71], while the remaining 11 were newly formulated (although based, in most cases, on observations made in earlier works). We scored all taxa in the matrix compiled by Longrich et al. [42] for these 25 additional characters, and rescored these taxa as necessary for the eight modified characters. Sources used for scoring these taxa are listed in the Supporting Information (Table S3 in File S1). The complete character list is provided as Appendix S1 in File S1.

#### Taxa

For most taxa, we maintained Longrich et al. 's [42] scores for the 197 characters that remained unaltered from their analysis. We did, however, update these authors' scores for the Mongolian oviraptorid *Khaan mckennai* based on information provided in the monographic description of this species recently published by Balanoff and Norell [72]. The resulting score changes are summarized in Table S4 in File S1.

Furthermore, in their taxonomic revision of Caenagnathidae, Longrich et al. [42] referred a large number of fragmentary, often isolated fossils from the Campanian of western Canada to three species: *Caenagnathus collinsi*, *Chirostenotes pergracilis*, and *Leptorhynchos elegans*. Nevertheless, many of these referred specimens preserve no skeletal elements in common with unambiguous materials of the species to which they were assigned; consequently, we regard these referrals with caution. An in-depth taxonomic reassessment of Campanian caenagnathids is beyond the scope of the present paper. For the current phylogenetic analysis, however, we took what we believe to be a more conservative approach in evaluating the affinities of these taxa: we rescored *C*. *collinsi*, *C*. *pergracilis*, and ‘*L*.’ *elegans* (as *Elmisaurus elegans*, as proposed by Currie [73]) using only their respective holotypes and any uncontroversially referred material that includes overlapping elements. A summary of these specimens is provided in Table S5 in File S1, and the resulting scoring changes are enumerated in Table S6 in File S1. Sources used for character information on these taxa are as in Table S3 in File S1.

We then added three operational taxonomic units (OTUs) to the analysis to encompass specimens we removed from *C*. *collinsi*, *C*. *pergracilis*, and *E*. *elegans*, respectively. Namely, these were *Macrophalangia canadensis* (for CMN 8538, the holotype of *M*. *canadensis*, a distal portion of a hind limb that Longrich et al. [42] referred to *C*. *collinsi*), ‘*Caenagnathus*’ *sternbergi* (for the holotype of this species [CMN 2690] and other mandibular material that Longrich et al. [42] referred to *C*. *pergracilis*), and an OTU that we term ‘Alberta dentary morph 3’ (for dentaries that Longrich et al. [42] referred to *E*. *elegans*). (We term this last OTU ‘Alberta dentary morph 3’ to differentiate it from the other two caenagnathid dentary morphotypes known from the Campanian of western Canada, those of *C*. *collinsi* and ‘*C*.’ *sternbergi*.) A summary of the specimens and sources used to score each of these three added OTUs is provided in Table S7 in File S1.

Finally, we added ten oviraptorosaurian species to the matrix: Banji long, Caudipteryx dongi, Ganzhousaurus nankangensis, Jiangxisaurus ganzhouensis, Nankangia jiangxiensis, Ojoraptorsaurus boerei, Shixinggia oblita, Similicaudipteryx yixianensis, Wulatelong gobiensis, and Yulong mini. Five of these taxa (C. dongi, J. ganzhouensis, O. boerei, S. yixianensis, and W. gobiensis) had never, to our knowledge, been incorporated into a numerical phylogenetic analysis before (although, in the cases of J. ganzhouensis and W. gobiensis, this is likely due to the fact that known material of each has only recently been published [74], [75]). Sources of character information for each of these ten taxa are detailed in Table S8 in File S1.

The complete phylogenetic data matrix is provided as Appendix S2 in File S1. Annotated .nex and .tnt files are also available from the senior author (M.C.L.) upon request.

#### Analysis

**Figure 6:**
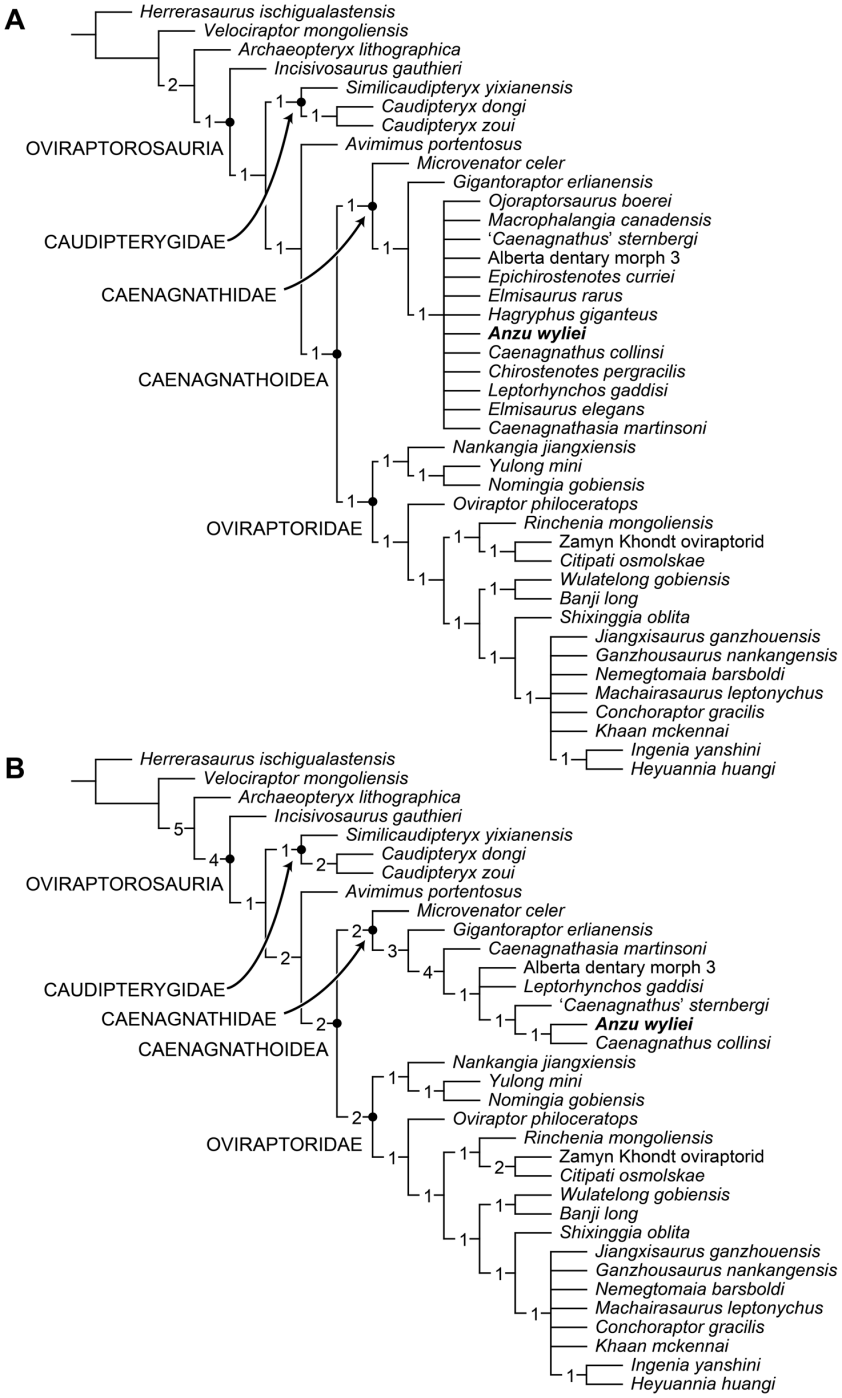
Strict consensus trees resulting from successive trials of phylogenetic analysis. Numbers adjacent to each node are Bremer support values; named nodes are indicated with black dots. (**A**) Strict consensus of 2,610 most parsimonious trees of 509 steps recovered by initial analysis of all 41 taxa (38 oviraptorosaurs) included in the matrix. (**B**) Strict consensus of seven most parsimonious trees of 498 steps resulting from an analysis of 34 taxa (31 oviraptorosaurs), excluding all members of Caenagnathidae (as recovered by the initial trial) for which definitive mandibular material has not yet been discovered.

The matrix of 41 taxa (38 oviraptorosaurs) and 230 osteological characters was analyzed using TNT (Tree Analysis Using New Technology) version 1.1 (Willi Hennig Society Edition) [76]. A traditional search (tree bisection-reconnection swapping algorithm, 1,000 random seeds, 1,000 replicates, 10 trees saved per replication) yielded 2,610 most parsimonious trees of 509 steps. The strict consensus of these trees (Figure 6A) supports the monophyly of several subclades within Oviraptorosauria, including Caudipterygidae, Caenagnathoidea, Oviraptoridae, and Caenagnathidae. *Anzu* is recovered as a derived caenagnathid, along with a large number of poorly known Late Cretaceous forms from North America as well as the Asian taxa *Caenagnathasia* and *Elmisaurus rarus*. Interestingly, as proposed by Longrich et al. [42], the Early Cretaceous *Microvenator* and the enormous *Gigantoraptor* are positioned as basal caenagnathids. This contrasts with the results of most previous studies, in which these genera have been regarded as a probable basal (i.e., non-caenagnathoid) oviraptorosaur [1], [60] and an oviraptorid [4], respectively.

In all trees, the clade Caenagnathoidea is supported by the following synapomorphic character states: palatal shelf of maxilla with two longitudinal ridges and tooth-like ventral process (character 11, state 1); pneumatic quadrate (character 45, state 1); dentary extremely short and deep, with maximum depth 50% or more of length (character 78, state 2); mandibular articular facet for quadrate formed exclusively of articular (character 90, state 1); cervical ribs of adults loosely attached to respective vertebrae (character 104, state 0); lateral pneumatic fossae present in caudal centra, at least in anterior part of tail (character 113, state 1); arched iliac dorsal margin (character 136, state 1); mesopubic (i.e., subvertically oriented) pubis (character 145, state 1); pubic shaft concave anteriorly (character 146, state 1); anterior and greater trochanters in contact (character 150, state 1); well-developed adductor fossa and associated anteromedial crest on distal femur (character 153, state 1); ratio of maximum length of metatarsus to that of femur 0.4–0.6 (character 160, state 0); posteroventrally directed retroarticular process (character 198, state 1); and lateral ridge of femur absent or represented by faint rugosity (character 213, state 0). Caenagnathidae is supported by the following synapomorphies: preacetabular process expanded ventrally well below level of dorsal acetabular margin (character 138, state 1); lateral surface of dentary bearing deep fossa, sometimes with associated pneumatopore (character 167, state 1); and ischial peduncle of pubis with prominent medial fossa (character 201, state 1). The unnamed node comprising *Gigantoraptor* plus more derived caenagnathids is supported by the following morphologies: fused mandibular symphysis (character 73, state 2); ratio of length of radius to length of humerus 0.8 or less (character 126, state 0); ventral symphyseal process of dentary absent (character 165, state 0); and humeral shaft strongly bowed laterally (character 226, state 1).

**Figure 7:**
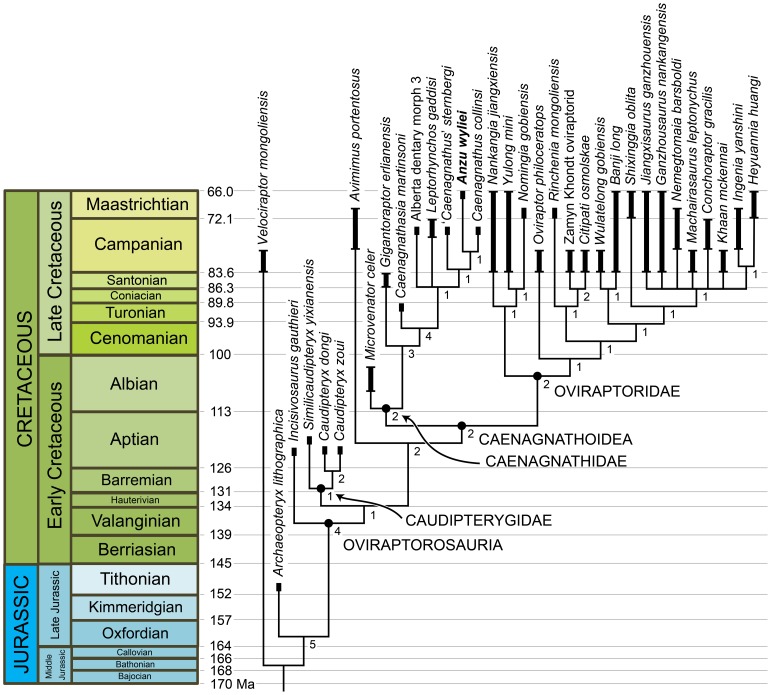
Calibrated phylogeny of oviraptorosaurian theropods showing hypothesized position of *Anzu wyliei* gen. et sp. nov. Depicted topology is the strict consensus of seven most parsimonious trees of 498 steps resulting from an analysis of 34 taxa (31 oviraptorosaurs) scored for 230 morphological characters (Figure 6B). Numbers adjacent to each node are Bremer support values; named nodes are indicated with black dots. Thick black bars indicate stratigraphic ranges of each taxon; small crossbars at ends of some bars indicate taxa that are especially poorly stratigraphically constrained (e.g., most Asian Late Cretaceous forms). Time scale follows [98]. Sources for stratigraphic ranges of included taxa are provided in Table S9 in File S1.

In an attempt to achieve better phylogenetic resolution within Caenagnathidae, we conducted a second analytical trial that omitted all members of this clade (as recovered by our initial analysis) for which definitive mandibular material is still unknown (i.e., *C*. *pergracilis*, *E*. *elegans*, *E*. *rarus*, *Epichirostenotes*, *Hagryphus*, *Macrophalangia*, *Ojoraptorsaurus*). The resulting matrix of 34 taxa (31 oviraptorosaurs) was analyzed using the protocols described above, yielding seven most parsimonious trees of 498 steps. The strict consensus of these trees (Figures 6B, 7) provides improved resolution among derived Caenagnathidae; the remainder of the topology is identical to that recovered in our initial trial, though in many cases Bremer support values are higher (Figure 6). *Caenagnathasia* is recovered as the outgroup to a clade (Caenagnathinae *sensu* Longrich et al. [42]) comprised by several Campanian–Maastrichtian caenagnathids from North America: *Anzu*, *C*. *collinsi*, ‘*C*.’ *sternbergi*, *Leptorhynchos gaddisi*, and ‘Alberta dentary morph 3.’ Within this clade, there is a basal polytomy formed by *L*. *gaddisi*, ‘Alberta dentary morph 3,’ and a ‘*C*.’ *sternbergi*–*C*. *collinsi*–*Anzu* clade. Within the latter, ‘*C*.’ *sternbergi* is basal to *C*. *collinsi* plus *Anzu*.

In the seven trees recovered by this second trial, Caenagnathoidea is supported by most of the same character states as in the initial trial. Nevertheless, the following characters are no longer optimized as caenagnathoid synapomorphies: palatal shelf of maxilla with two longitudinal ridges and tooth-like ventral process (character 11, state 1); cervical ribs in adults loosely attached to respective vertebrae (character 104, state 0); and arched iliac dorsal margin (character 136, state 1). Caenagnathidae and *Gigantoraptor* plus more derived caenagnathids are supported by the same synapomorphies as in the initial trial. *Caenagnathasia* plus Caenagnathinae is supported by the following mandibular features: maximum depth of dentary between 25% and 50% of length (character 78, state 1); anterodorsal margin of dentary broadly concave in lateral view (character 84, state 2); lingual triturating shelf present (character 188, state 1); symphyseal ridges inside tip of beak present but weakly developed (character 189, state 1); lingual ridges inside lateral occlusal surface of beak present (character 190, state 1); and pneumatic dentaries (character 192, state 1). Caenagnathinae is supported by a single synapomorphy, symphyseal ridges inside tip of beak well developed (character 189, state 2), as is the ‘*C*.’ *sternbergi*–*C*. *collinsi*–*Anzu* clade: tip of dentary projecting anterodorsally (character 184, state 1). Lastly, the *C*. *collinsi* plus *Anzu* clade is supported by two characters of the dentary: symphyseal portion not downturned (character 75, state 0) and symphysis lacking hourglass-shaped ventral depression (character 186, state 0).

In sum, our phylogenetic results reaffirm caenagnathid monophyly (*contra* [31], [43]), and moreover suggest that this clade was considerably longer-lived and more morphologically diverse than previously appreciated. *Microvenator* and *Gigantoraptor* are postulated as the basalmost known members of the group, whereas *Anzu* is the probable sister-taxon of *Caenagnathus collinsi* within a derived caenagnathid clade that is thus far known only from the Late Cretaceous of western North America.

### Body mass estimation

We estimated the body mass of the holotype of *Anzu wyliei* (CM 78000) using the equation for mass estimation in bipedal dinosaurs generated by Anderson et al. [77]: W = 0.16*C_f_^2.73^, where W is body weight in g and C_f_ is minimum femoral circumference in mm. The minimum circumference of the right femur of CM 78000 is 169 mm, which yields an estimated body mass of 193 kg for this individual. Another recent study that employed Christiansen and Fariña's [78] equation for calculating theropod body mass on the basis of femoral length generated an estimate of 247.8 kg for *Anzu*, based on a cast skeleton reconstructed from CM 78000 and CM 78001 (CM 78003) [79]. Using this same equation (log_10_ *y* = −6.288+3.222*log_10_FL, where *y* is body mass in kg and FL is femoral length in mm) and the femoral length of CM 78000 (525 mm; see Table S2 in File S1) yields a mass estimate of 299.5 kg for this individual. Consequently, in life, the body mass of the holotype of *Anzu wyliei* was probably approximately 200–300 kg.

## Discussion

### Caenagnathid morphology

*Anzu wyliei* provides, for the first time, a nearly complete view of the skeletal morphology of a caenagnathid oviraptorosaur. With an approximate body length of 3.5 m, a height at the hip of roughly 1.5 m (Figures 4A, 5A), and a body mass of some 200–300 kg, *Anzu* is among the largest known oviraptorosaurs, second in size only to its probable close relative *Gigantoraptor* (the mass of which has been estimated at between 1,400 and 3,246 kg [4], [79]). Moreover, when considered in light of very small-bodied taxa such as *Caenagnathasia* (with an estimated mass of 5 kg [46]) and *Elmisaurus elegans* [73], it appears highly likely that caenagnathids encompassed a much greater range of body sizes than did other oviraptorosaurs, and indeed, many other non-avian theropod groups. Further studies of caenagnathid growth and the ontogenetic status of individual specimens belonging to this clade are needed to evaluate this hypothesis.

The skull of *Anzu* is deep and narrow, as previously noted for *Epichirostenotes* [40], and is crowned by a tall, cassowary-like crest. Surprisingly, the jugal resembles those of non-oviraptorosaurian theropods in being dorsoventrally deep and posteriorly bifid. The jaws are edentulous, and, as in other derived caenagnathids, the occlusal surface of the coossified dentaries is characterized by a complex array of ridges and grooves. With 12 cervical vertebrae, the neck is long, but it is also transversely wide across the cervical ribs, especially toward its posterior end. The tail is extensively pneumatized and terminates in a short sequence of highly modified vertebrae that collectively comprise a pygostyle-like structure. The sternal plates closely resemble those of oviraptorids in having a pair of well-developed lateral processes [18]. The distal end of the radius is peculiar in being divided into a pair of tuberosities. The manus is proportionally large, and, like those of other derived caenagnathids, its unguals exhibit proximodorsal ‘lips’ that are more prominent than in most other oviraptorosaurs. Although incompletely known in *Anzu*, manual digits II and III were probably elongate, as in other derived caenagnathids in which the manus is better known (e.g., *Chirostenotes pergracilis*, *Elmisaurus rarus*, *Hagryphus*) [34], [39], [63], [64]. The pubic shaft is straight and the ischium is short and distinctly curved. The hind limb elements are gracile, with the tibia being substantially longer than the femur. The pes is incompletely preserved in *Anzu*, but what is known suggests that its digits were elongate, as in other derived caenagnathids (e.g., *Macrophalangia* [35]).

### Paleoecology of caenagnathidae

The mode of life of caenagnathids and other oviraptorosaurs has been the subject of much speculation. The fact that the jaws of most oviraptorosaurs lack teeth and were probably covered by a keratinous rhamphotheca has frustrated attempts to infer the diets of these theropods. The earliest suggestion was that the oviraptorid *Oviraptor philoceratops* fed on the eggs of other dinosaurs, based on the association of the holotypic skeleton with a clutch of eggs that was, at the time, assigned to the ceratopsian *Protoceratops* [15]. Eggs of this morphotype were subsequently identified as those of oviraptorids [80], and additional oviraptorid skeletons were found, often in bird-like brooding positions, atop nests of such eggs [16]–[18], [20]. Consequently, the prevailing view is that, rather than preying on the nests of other dinosaurs, these oviraptorids were guarding their own nests at the time of death. Nevertheless, the likelihood that oviraptorosaurs brooded their nests does not preclude the possibility that these theropods may also have eaten the eggs of other vertebrates. Indeed, Currie et al. [46] likened the tooth-like ventral processes of the palatal shelves of oviraptorid maxillae to the egg-cracking vertebral processes of the extant egg-eating snake *Dasypeltis scabra*; based in part on this similarity, these authors argued that oviraptorosaurs may have subsisted on eggs and small-bodied vertebrates. The discovery of two embryonic or perinatal skulls of the troodontid theropod *Byronosaurus* in a nest with oviraptorid eggs [80], [81] may support this view, in that it suggests that these young troodontids may have been captured and killed by the adult oviraptorid that was presumably tending this nest. Nevertheless, this conclusion should be regarded as tentative, given that, according to J. Clark (pers. comm.), there are no signs of predation on the *Byronosaurus* specimens. Furthermore, this nest comes from one of the most densely fossiliferous localities within the Upper Cretaceous Djadokhta Formation, with dozens of lizard, dinosaur, and mammal specimens found in close proximity [80], [81]. As such, it is possible that the association of these troodontid skulls with this oviraptorid nest is due to taphonomic or preservational factors rather than representing an authentic paleoecological interaction.

Other previous works have offered alternative dietary hypotheses for Oviraptorosauria. Barsbold [82], [83] has suggested that the edentulous but robust jaws of oviraptorids were employed to crush mollusks; nevertheless, this proposal has been strongly challenged on anatomical, biomechanical, and paleoecological grounds [46], [49]. Cracraft [38] and Funston and Currie [49] noted the close structural similarities between caenagnathid mandibles and those of dicynodontian synapsids, the latter of which are considered largely or exclusively herbivorous. In both caenagnathids and dicynodonts, the morphology of the mandibular joint indicates extensive fore-and-aft motion of the mandible, which could have facilitated the slicing of plant fodder between the opposing, beak-covered jaws. Based on inferences concerning jaw mechanics, Smith [25] also proposed that *Oviraptor* and its relatives may have been herbivores, whereas Longrich et al. [42] recently came to the same conclusion on the basis of observed anatomical convergences between caenagnathid mandibles and those of extant herbivorous turtles and birds.

In their recent description of the well-preserved Campanian caenagnathid mandible TMP 2001.012.0012, Funston and Currie [49] conducted some of the most detailed comparisons to date of the jaws of these oviraptorosaurs with those of dicynodonts. They observed that TMP 2001.012.0012 possesses four of the five mandibular features that King et al. [84] regarded as specializations for more efficient shearing of vegetation in these synapsids. Specifically, these are a jaw joint that permits anteroposterior movement of the mandible, an intramandibular fenestra, loss of the coronoid eminence coupled with posterodorsal expansion of the dentary, and a fused dentary symphysis. Interestingly, in addition to these four characters, *Anzu* and *Gigantoraptor* possess the fifth feature as well: a lateral flange on the dentary. King et al. [84] interpreted this structure as the area of insertion for anteriorly positioned adductor musculature in dicynodonts, and it may have served the same purpose in these two caenagnathids. Regardless of its functional significance, the presence of a lateral flange on the dentaries of *Anzu* and *Gigantoraptor* indicates that the degree of convergence in mandibular anatomy between Caenagnathidae and Dicynodontia was even greater than previously appreciated.

Intriguingly, what is perhaps the most compelling evidence for oviraptorosaurian herbivory has thus far been documented only in archaic, tooth-bearing members of the clade. Several specimens of *Caudipteryx* have been preserved with clusters of gastroliths [2], [8], [26], which strongly suggests the presence of a gastric mill in this taxon [85], and the anteriormost premaxillary teeth of *Incisivosaurus* resemble rodent incisors [5]. Furthermore, the only known specimen of the recently described basal oviraptorosaur *Ningyuansaurus* preserves numerous ovate structures within the body cavity that may be seeds [7]. Perhaps, as suggested by Zanno and Makovicky [85], basal oviraptorosaurs were wholly or predominantly herbivorous, but the evolution of a keratinous beak in a derived subset of the clade (*Avimimus* plus Caenagnathoidea according to our phylogenetic results) led to a broader array of diets among members of this group, including predation on small animals and/or eggs in addition to herbivory.

The behavioral implications of the distinctive appendicular skeletal anatomy of Caenagnathidae have also been a topic of frequent discussion in the literature. Currie and Russell [39] commented on the proportionally long hind limbs and large, broad pedes of these oviraptorosaurs, and proposed that caenagnathids may have been specialized waders that hunted freshwater invertebrates. These authors further suggested that the slender third manual digit of *Chirostenotes pergracilis* may have been used for extricating small prey from crevices in stream bottoms or trees [39], [45]. More recently, Senter and Parrish [48] found the structure and range of inferred motion of the manus of *C*. *pergracilis* to be compatible with a hooking function. They also proposed that the manus was useful for crevice probing, but that, rather than manual digit III, it was the long second manual digit, with its straighter ungual, that served this purpose.

Various authors have provided still other interpretations of the functional morphology of caenagnathid limbs. Based on the length and proportions of the hind limb, Currie [45] suggested that these oviraptorosaurs may have been quick, agile cursors. Varricchio [47], by contrast, compared the phalangeal proportions of a small caenagnathid pes from the Hell Creek Formation of Montana (MOR 752) to those of several modern bird species, and concluded that the pedal digits of these oviraptorosaurs were better adapted for grasping than for running. On this basis he hypothesized that caenagnathids may have used their feet for climbing or prey capture. Longrich et al. [42] also proposed that caenagnathids might have been semi-arboreal.

The depositional environments in which caenagnathid fossils have been found may provide additional clues to the habitat preferences of these theropods. An apparent paleoenvironmental distinction between caenagnathids and oviraptorids has long been noted [1], [42], [46]: namely, whereas the vast majority of oviraptorid fossils have been recovered from rocks interpreted to represent arid to semi-arid settings [20], [86]–[89], most caenagnathids have been discovered in fluvial sediments that are thought to have been deposited under more mesic conditions [90]–[92]. As such, caenagnathids are thought to have been adapted to wetter, more humid surroundings than were their oviraptorid relatives.

The sedimentology of the localities in the Hell Creek Formation that have yielded associated caenagnathid specimens may provide more specific insights into the types of environments that these animals frequented [93]. The more complete CM specimens of *Anzu* were recovered from silty mudstones that are herein interpreted as overbank sediments, whereas the fragmentary MRF 319 was found in a channel lag deposit. A fourth associated caenagnathid skeleton from the Hell Creek Formation, discovered in 2013 by the Burpee Museum of Natural History, was also preserved in an organic-rich mudstone (S. Williams, pers. comm.; T.R.L., pers. obs.). The lithology of the site that yielded the associated pes MOR 752 was not described [47]. Consequently, the three most complete caenagnathid skeletons recovered from the Hell Creek Formation to date have all come from mudstones that are interpreted as low-energy overbank deposits. Given the limited sample of Hell Creek oviraptorosaurian fossils at present, this pattern could be the result of taphonomic filtering (as has been documented in other North American Late Cretaceous continental units, e.g., [94]), with the comparatively small and delicate (relative to those of most other Hell Creek non-avian dinosaurs) bones of oviraptorosaurs being scattered or destroyed in higher-energy fluvial systems. Nevertheless, the possible association of caenagnathid skeletons with overbank deposits might instead reflect an authentic paleoecological preference comparable to that hypothesized for other late Maastrichtian North American dinosaurs [93]. Namely, in the paleoenvironment represented by the Hell Creek Formation, these oviraptorosaurs may have favored floodplain habitats over those closer to river margins.

To our knowledge, ichnological evidence has not yet been brought to bear on the issue of caenagnathid paleoecology; nevertheless, fossil trackways may offer additional insights into the habits of these dinosaurs. The distinctive theropod ichnotaxon *Saurexallopus* spp. from the Maastrichtian of the Rocky Mountain region of the United States [95], [96] was recently identified as having been made by one or more, emu- to ostrich-sized, *Chirostenotes*-like oviraptorosaurian taxa with estimated hip heights of 1.1–1.75 m [97]. The pedal morphology, stratigraphic and geographic provenance, and inferred size range of the *Saurexallopus* trackmaker(s) thus corresponds closely with known body fossils of *Anzu*, and as such, it is conceivable that footprints of this type may have been made by *A*. *wyliei* and/or other, closely related caenagnathids. Consequently, the depositional settings of *Saurexallopus* trackways may shed light on the habitat preferences of latest Cretaceous caenagnathids in western North America. The only known specimens of the type ichnospecies *S*. *lovei*, from the middle Maastrichtian Harebell Formation of northwestern Wyoming, were made by at least two individual theropods that walked over bioturbated tidal flats and into shallow waters along the coast of a nearshore marine or brackish embayment [95]. Tracks of the second named ichnospecies, *S*. *zerbsti*, were discovered in a silty, fine-grained sandstone of the upper Maastrichtian Lance Formation of northeastern Wyoming that has been interpreted to represent an ephemeral pond [96]. Interestingly, in both cases, the depositional environments of the *Saurexallopus* tracks indicate that these putative caenagnathids entered shallow bodies of water, a circumstance that is consistent with the wading hypothesis offered by Currie and Russell [39] exclusively on the basis of body fossils.

To conclude, a considerable range of possible lifestyles has been proposed for Caenagnathidae, some of which may be mutually exclusive (e.g., strict herbivory versus predation on small vertebrates; wading versus climbing). This is in spite of the fact that, prior to the discovery of *Anzu wyliei*, our knowledge of the anatomy of these theropods was mostly limited to bones of the mandible, manus, and pes. Although the morphology of the new Hell Creek caenagnathid does not provide definitive evidence for choosing between any of these various paleoecological proposals, some hypotheses appear more plausible than others. For instance, if, as in caudipterygids (the only oviraptorosaurs for which forelimb integument has been extensively preserved [2], [9], [26]), manual digit II of caenagnathids supported quill feathers, the crevice-probing function proposed for this digit by Senter and Parrish [48] is difficult to envision. Furthermore, it seems unlikely that large-bodied caenagnathids such as *Anzu* and *Hagryphus* would have spent much time in trees, at least as adults (*contra* [47]). On the other hand, the jaw morphology of caenagnathids does suggest that these theropods were capable of processing a variety of potential food items. Furthermore, evidence from the depositional settings of caenagnathid body and possible trace fossils suggests that these theropods may have favored well-watered floodplain habitats over drier environs. In sum, in our view, *Anzu* and other derived caenagnathids may well have been ecological generalists that fed upon vegetation, small animals, and perhaps even eggs on the humid coastal plains of western North America at the end of the Age of Dinosaurs.

## Supporting Information

File S1 **Supporting Information.** Including the following: (1) abbreviations of institutions cited in Supporting Information; (2) inventory of North American Late Cretaceous oviraptorosaur specimens (Table S1); (3) measurements of *Anzu wyliei* gen. et sp. nov. (Table S2); (4) phylogenetic methods (including Tables S3–S9 and Appendices S1 and S2); and (5) references cited in Supporting Information. (PDF)
(DOC)
