# Correction: A New Large-Bodied Oviraptorosaurian Theropod Dinosaur from the Latest Cretaceous of Western North America

**DOI:** 10.1371/journal.pone.0125843

**Published:** 2015-04-29

**Authors:** Matthew C. Lamanna, Hans-Dieter Sues, Emma R. Schachner, Tyler R. Lyson

The first sentence of the Acknowledgments is incorrect. The correct statement should read: “We thank Nuss Fossils for discovering CM 78000 and CM 78001 and the field area that yielded these specimens. We also acknowledge Triebold Paleontology, Inc. for the preparation of these skeletons.”
