# Supplementary material for: A New Large-Bodied Oviraptorosaurian Theropod Dinosaur from the Latest Cretaceous of Western North America
Source: PLoS One. 2014 Mar 19;9(3):e92022. doi: 10.1371/journal.pone.0092022 (PMC3960162; doi:10.1371/journal.pone.0092022)
Supplement: File S1 — Supporting Information. Including the following: (1) abbreviations of institutions cited in Supporting Information; (2) inventory of North American Late Cretaceous oviraptorosaur specimens (Table S1); (3) measurements of Anzu wyliei gen. et sp. nov. (Table S2); (4) phylogenetic methods (including Tables S3–S9 and Appendices S1 and S2); and (5) references cited in Supporting Information. (PDF) (DOC) [file pone.0092022.s001.doc]

**File S1 Supporting Information**

**for**

**A New Large-Bodied Oviraptorosaurian Theropod Dinosaur from the Latest Cretaceous of Western North America**

**Matthew C. Lamanna^1^*, Hans-Dieter Sues^2^, Emma R. Schachner^3^, Tyler R. Lyson^4^**

**1** Section of Vertebrate Paleontology, Carnegie Museum of Natural History, Pittsburgh, Pennsylvania, United States of America,

**2** Department of Paleobiology, National Museum of Natural History, Smithsonian Institution, Washington, District of Columbia, United States of America,

**3** Department of Biology, University of Utah, Salt Lake City, Utah, United States of America,

**4** Department of Vertebrate Zoology, National Museum of Natural History, Smithsonian Institution, Washington, District of Columbia, United States of America

___________________

*E-mail: [lamannam@carnegiemnh.org](mailto:lamannam@carnegiemnh.org)

**Contents**

| (1) Abbreviations of institutions cited in Supporting Information | 3 |
| --- | --- |
| (2) Inventory of North American Late Cretaceous oviraptorosaur specimens (Table S1) | 4 |
| (3) Measurements of *Anzu wyliei* gen. et sp. nov. (Table S2) | 6 |
| (4) Supplementary information on phylogenetic analysis (Tables S3–S9; Appendices S1, S2) | 12 |
| (5) References cited in Supporting Information | 63 |

**(1) Abbreviations of institutions cited in Supporting Information**

BHM, Black Hills Institute of Geological Research, Hill City, South Dakota, United States of America; CM, Carnegie Museum of Natural History, Pittsburgh, Pennsylvania, United States of America; CMN, Canadian Museum of Nature, Ottawa, Ontario, Canada; FMNH, Field Museum of Natural History, Chicago, Illinois, United States of America; MOR, Museum of the Rockies, Bozeman, Montana, United States of America; MRF, Marmarth Research Foundation, Marmarth, North Dakota, United States of America; ROM, Royal Ontario Museum, Toronto, Ontario, Canada; SDSM, South Dakota School of Mines and Technology, Rapid City, South Dakota, United States of America; SMP, State Museum of Pennsylvania, Harrisburg, Pennsylvania, United States of America; TMM, University of Texas Memorial Museum, Austin, Texas, United States of America; TMP, Royal Tyrrell Museum of Palaeontology, Drumheller, Alberta, Canada; UMNH, Natural History Museum of Utah, Salt Lake City, Utah, United States of America; UTEP, University of Texas-El Paso, El Paso, Texas, United States of America.

**(2) Inventory of North American Late Cretaceous oviraptorosaur specimens**

**Table S1. Oviraptorosaurian body fossils from the Late Cretaceous of North America that have been identified in the scientific literature (listed alphabetically by specimen number).** Specimens that have been mentioned or illustrated but that for which no specimen number has been provided (e.g., those listed by ] and those figured by ] and Bartlett [2004]) are not included. Abbreviation: S–A–C, surangular–articular–coronoid complex.

| **Specimen** | **Elements** | **Formation** | **Province/**  **state** | **Age** | **Originally described by** | **Holotype of** |
| --- | --- | --- | --- | --- | --- | --- |
| CM 78000 | Largely complete skeleton | Hell Creek | South Dakota | Maastrichtian (late) | This paper | *Anzu wyliei* |
| CM 78001 | Largely complete skeleton | Hell Creek | South Dakota | Maastrichtian (late) | This paper | N/A |
| CMN 12322 | Manual ungual II | Dinosaur Park | Alberta | Campanian (late) |  | N/A |
| CMN 2367 | Both articulated manus | Dinosaur Park | Alberta | Campanian (late) |  | *Chirostenotes pergracilis* |
| CMN 2690 | Partial S–A–C | Dinosaur Park | Alberta | Campanian (late) |  | ‘*Caenagnathus*’ *sternbergi* |
| CMN 8504 | Dorsal & caudal vertebrae | ? | Saskatchewan | Maastrichtian (late) |  | N/A |
| CMN 8538 | Distal hind limb | Dinosaur Park | Alberta | Campanian (late) |  | *Macrophalangia canadensis* |
| CMN 8776 | Nearly complete mandible | Dinosaur Park | Alberta | Campanian (late) |  | *Caenagnathus collinsi* |
| CMN 9570 | Metatarsal II | Dinosaur Park | Alberta | Campanian (late) |  | N/A |
| FMNH PR 2296  (= BHM 2033) | Nearly complete S–A–C | Hell Creek | South Dakota | Maastrichtian (late) |  | N/A |
| MOR 1107 | Partial S–A–C | Two Medicine | Montana | Campanian |  | N/A |
| MOR 752 | Partial pes | Hell Creek | Montana | Maastrichtian (late) |  | N/A |
| MRF 319 | Partial postcranial skeleton | Hell Creek | North Dakota | Maastrichtian (late) | This paper | N/A |
| ROM 37163 | Partial metatarsal II | Dinosaur Park | Alberta | Campanian (late) |  | N/A |
| ROM 43250 | Partial skeleton | Horseshoe Canyon | Alberta | Campanian (late)–  Maastrichtian (early) |  | *Epichirostenotes curriei* |
| ROM 781 | Partial tarsometatarsus | Dinosaur Park | Alberta | Campanian (late) |  | *Elmisaurus elegans* |
| SDSM 64385 | Manual ungual | Hell Creek | South Dakota | Maastrichtian (late) |  | N/A |
| SMP VP-1458 | Pubes | Ojo Alamo | New Mexico | Maastrichtian (early) |  | *Ojoraptorsaurus boerei* |
| TMM 42335-40 | Caudal vertebra | Aguja | Texas | Campanian (late) |  | N/A |
| TMM 42920-2 | Manual ungual II | Aguja | Texas | Campanian (late) |  | N/A |
| TMM 43057-354 | Distal metatarsal III | Aguja | Texas | Campanian (late) |  | N/A |
| TMM 43057-357 | Pedal ungual | Aguja | Texas | Campanian (late) |  | N/A |
| TMM 43057-36 | Proximal metatarsal IV | Aguja | Texas | Campanian (late) |  | N/A |
| TMM 45920-1 | Dentaries | Aguja | Texas | Campanian (late) |  | *Leptorhynchos gaddisi* |
| TMP 1975.011.0033 | Cervical vertebra | Dinosaur Park | Alberta | Campanian (late) |  | N/A |
| TMP 1979.008.0622 | Partial dentaries | Dinosaur Park | Alberta | Campanian (late) |  | N/A |
| TMP 1979.014.0499 | Manual ungual II | Dinosaur Park | Alberta | Campanian (late) |  | N/A |
| TMP 1979.020.0001 | Partial postcranial skeleton | Dinosaur Park | Alberta | Campanian (late) |  | N/A |
| TMP 1982.016.0006 | Metatarsal | Dinosaur Park | Alberta | Campanian (late) |  | N/A |
| TMP 1982.039.0004 | Proximal tarsometatarsus | Dinosaur Park | Alberta | Campanian (late) |  | N/A |
| TMP 1984.163.0102 | Synsacrum | Dinosaur Park? | Alberta | Campanian (late)? |  | N/A |
| TMP 1990.056.0006 | Nearly complete dentaries | Dinosaur Park | Alberta | Campanian (late) |  | N/A |
| TMP 1991.144.0001 | Nearly complete dentaries | Dinosaur Park | Alberta | Campanian (late) |  | N/A |
| TMP 1992.036.0053 | Caudal vertebra | Dinosaur Park | Alberta | Campanian (late) |  | N/A |
| TMP 1992.036.0390 | Nearly complete dentaries | Dinosaur Park | Alberta | Campanian (late) |  | N/A |
| TMP 1992.036.0448 | Manual or pedal phalanx | Dinosaur Park | Alberta | Campanian (late) |  | N/A |
| TMP 1992.040.0044 | Partial dentaries | Dinosaur Park | Alberta | Campanian (late) |  | N/A |
| TMP 1996.012.0141 | Metatarsal | Dinosaur Park | Alberta | Campanian (late) |  | N/A |
| TMP 2001.012.0012 | Nearly complete mandible | Dinosaur Park | Alberta | Campanian (late) |  | N/A |
| UMNH VP 12765 | Distal forelimb, partial pes | Kaiparowits | Utah | Campanian (late) |  | *Hagryphus giganteus* |
| UTEP B38 L-3 | Femur | Aguja | Texas | Campanian (late) |  | N/A |

**(3) Measurements of *Anzu wyliei* gen. et sp. nov.**

**Table S2. Selected measurements (mm) of specimens of *Anzu* *wyliei* gen. et sp. nov.** Measurements of dimensions greater than 205 mm were taken with a tape measure (rather than digital calipers) and are therefore considered slightly less precise; consequently, such measurements are provided only to the nearest 5 mm. Abbreviations: aofe, antorbital fenestra; ^L^, left; ^R^, right; *, element incomplete, measurement as preserved; ^†^, element incomplete, measurement estimated.

| **Element/dimension** | **CM 78000** | **CM 78001** | **MRF 319** |
| --- | --- | --- | --- |
| **Skull and mandible** |  |  |  |
| *Premaxilla* |  |  |  |
| Length, anteroposterior | N/A | 250^L^* | N/A |
| Length of tomial (ventral) margin, anteroposterior | N/A | 68.0^L†^ | N/A |
| Height, dorsoventral | N/A | 320^L^* | N/A |
|  |  |  |  |
| *Maxilla* |  |  |  |
| Length, anteroposterior | N/A | 193.0^L^* | N/A |
| Height, dorsoventral | N/A | 210^L^* | N/A |
| Height of body at anterior end of aofe, dorsoventral | N/A | 69.0^L†^ | N/A |
|  |  |  |  |
| *Jugal* |  |  |  |
| Length, anteroposterior | N/A | 159.3^L^* | N/A |
| Height at posterior end, dorsoventral | N/A | 34.9^L^* | N/A |
|  |  |  |  |
| *Braincase* |  |  |  |
| Height, occipital condyle midline, dorsoventral | 10.4 | 12.3 | N/A |
| Width of occipital condyle, transverse | 20.9 | 21.7 | N/A |
| Height of foramen magnum, dorsoventral | 18.0 | 15.3 | N/A |
| Width of foramen magnum, transverse | 15.1 | 13.1 | N/A |
|  |  |  |  |
| *Dentary* |  |  |  |
| Length, anteroposterior | 175.5^L^* | N/A | N/A |
| Height at posterior end, dorsoventral | 51.5^L^ | N/A | N/A |
| Width, lateral shelf–sagittal midline | 38.5^L^* | N/A | N/A |
|  |  |  |  |
| *Surangular–articular–coronoid complex* |  |  |  |
| Length, anteroposterior | 192.6^L^* | N/A | N/A |
| Length of mandibular glenoid, anteroposterior | 35.7^L^, 35.0^R^ | N/A | N/A |
| Width of mandibular glenoid, mediolateral | 33.6^L^, 31.7^R^* | N/A | N/A |
| Length of retroarticular process, anteroposterior | 34.3^L^ | N/A | N/A |
| Height, base of retroarticular process, dorsoventral | 24.3^L^ | N/A | N/A |
|  |  |  |  |
| *Angular* |  |  |  |
| Length, anteroposterior | 145.6*^L^ | N/A | N/A |
| Height at anterior end, dorsoventral | 16.9*^L^ | N/A | N/A |
| Height at posterior end, dorsoventral | 23.0*^L^ | N/A | N/A |
|  |  |  |  |
| **Cervical vertebrae and ribs** |  |  |  |
| *Cervical 9* |  |  |  |
| Length of centrum, anteroposterior | N/A | 82.9^†^ | 100.5* |
| Length across zygapophyses, anteroposterior | N/A | 133.9 | 118.4* |
| Width across cervical ribs, transverse | N/A | 86.8^†^ | 132.5* |
| Height at posterior end, dorsoventral | N/A | 69.8^†^ | 70.3* |
|  |  |  |  |
| *Cervical 11* |  |  |  |
| Length of centrum, anteroposterior | N/A | 78.5^†^ | 89.6* |
| Length across zygapophyses, anteroposterior | N/A | 113.5* | 101.4* |
| Width across cervical ribs, transverse | N/A | 94.0^†^ | 113.6* |
| Height at posterior end, dorsoventral | N/A | 72.8^†^ | 93.8* |
|  |  |  |  |
| *Cervical 12* |  |  |  |
| Length of centrum, anteroposterior | N/A | 66.4* | 72.3* |
| Length across zygapophyses, anteroposterior | N/A | 90.6* | 98.0* |
| Width across cervical ribs, transverse | N/A | 120.9* | 148.9* |
| Height at posterior end, dorsoventral | N/A | 94.6* | 108.9* |
|  |  |  |  |
| **Dorsal vertebrae** |  |  |  |
| *Anterior dorsal* |  |  |  |
| Length of centrum, anteroposterior | N/A | 47.0 | N/A |
| Width of anterior end of centrum, transverse | N/A | 52.7 | N/A |
| Height of anterior end of centrum, dorsoventral | N/A | 47.8 | N/A |
| Width of posterior end of centrum, transverse | N/A | 35.6 | N/A |
| Height of posterior end of centrum, dorsoventral | N/A | 45.1 | N/A |
| Width across transverse processes, transverse | N/A | 120.4 | N/A |
| Height, dorsoventral | N/A | 142.1* | N/A |
|  |  |  |  |
| *Posterior dorsal* |  |  |  |
| Length of centrum, anteroposterior | N/A | 46.3* | N/A |
| Width of anterior end of centrum, transverse | N/A | 53.9* | N/A |
| Height of anterior end of centrum, dorsoventral | N/A | 62.9 | N/A |
| Width of posterior end of centrum, transverse | N/A | 64.1 | N/A |
| Height of posterior end of centrum, dorsoventral | N/A | 57.7 | N/A |
| Width across transverse processes, transverse | N/A | 120.5 | N/A |
| Height, dorsoventral | N/A | 196.4 | N/A |
|  |  |  |  |
| **Sacral vertebrae** |  |  |  |
| Length of sacrum, anteroposterior | N/A | 315 | N/A |
| Width of centrum 1, transverse | N/A | 66.1* | N/A |
| Height of centrum 1, transverse | N/A | 34.0* | N/A |
|  |  |  |  |
| **Caudal vertebrae** |  |  |  |
| *‘Pygal’ 1* |  |  |  |
| Length of centrum, anteroposterior | 33.9 | N/A | N/A |
| Width across transverse processes, transverse | 37.2^†^ | N/A | N/A |
| Height of anterior end, dorsoventral | 33.7 | N/A | N/A |
|  |  |  |  |
| *‘Pygal’ 2* |  |  |  |
| Length of centrum, anteroposterior | 37.2 | N/A | N/A |
| Width across transverse processes, transverse | 22.5 | N/A | N/A |
| Height of anterior end, dorsoventral | 39.0 | N/A | N/A |
|  |  |  |  |
| *‘Pygal’ 3* |  |  |  |
| Length of centrum, anteroposterior | N/A | N/A | N/A |
| Width across transverse processes, transverse | 17.1 | N/A | N/A |
| Height of anterior end, dorsoventral | 34.5 | N/A | N/A |
|  |  |  |  |
| **Pectoral girdle and forelimb** |  |  |  |
| *Scapula* |  |  |  |
| Length along curved lateral surface, dorsoventral | 415^R^ | N/A | N/A |
| Depth of posterior blade, anteroposterior | 49.0^L^, 44.8^R^* | N/A | 50.5^L^ |
|  |  |  |  |
| *Coracoid* |  |  |  |
| Length, dorsoventral | 121.0^R^ | N/A | 145.5^L^* |
|  |  |  |  |
| *Sternal plates* |  |  |  |
| Length, anteroposterior | N/A | 190.2^L^*, 194.2^R^ | N/A |
| Width across anterolateral process, mediolateral | N/A | 158.2^R^ | N/A |
| Width across xiphoid process, mediolateral | N/A | 181.3^R^ | N/A |
|  |  |  |  |
| *Humerus* |  |  |  |
| Length, proximodistal | 345^R^ | N/A | N/A |
| Width of proximal end, mediolateral | 70.1^R^ | N/A | N/A |
| Width of distal end, mediolateral | 62.3^R^ | N/A | N/A |
|  |  |  |  |
| *Radius* |  |  |  |
| Length, proximodistal | 280^L^ | N/A | 275^L^ |
| Width of proximal end, mediolateral | 30.3^L^ | N/A | 37.1^L^* |
| Width of distal end, mediolateral | 32.8^L^ | N/A | 36.1^L^* |
|  |  |  |  |
| *Ulna* |  |  |  |
| Length, proximodistal | 280^L^ | N/A | 280^L^ |
| Width of proximal end, mediolateral | 47.6^L^ | N/A | 42.6^L^* |
| Width of distal end, mediolateral | 45.0^L^ | N/A | 48.1^L^ |
|  |  |  |  |
| *Metacarpal II* |  |  |  |
| Length, proximodistal | 139.0 | N/A | N/A |
| Width of shaft, mediolateral | 17.8 | N/A | N/A |
|  |  |  |  |
| *Manual phalanx I-1* |  |  |  |
| Length, proximodistal | 123.5 | N/A | N/A |
| Width of shaft, mediolateral | 12.0 | N/A | N/A |
|  |  |  |  |
| *Manual phalanx I-2 (ungual)* |  |  |  |
| Length, proximodistal | 89.1 | N/A | N/A |
| Length along curved dorsal surface, proximodistal | 130.0 | N/A | N/A |
|  |  |  |  |
| *Manual phalanx II-1* |  |  |  |
| Length, proximodistal | 131.8 | N/A | N/A |
| Width of shaft, mediolateral | 17.4 | N/A | N/A |
|  |  |  |  |
| *Manual phalanx III-3* |  |  |  |
| Length, proximodistal | 82.8 | N/A | N/A |
| Height of shaft, dorsoventral | 11.9 | N/A | N/A |
| Width of shaft, mediolateral | 10.0 | N/A | N/A |
|  |  |  |  |
| **Pelvic girdle and hind limb** |  |  |  |
| *Pubis* |  |  |  |
| Length, proximodistal | N/A | 450^L^, 465^R^ | N/A |
| Length of pubic ‘apron,’ proximodistal | N/A | 191.3^L^, 203.4^R^ | N/A |
| Depth of proximal end, anteroposterior | N/A | 80.5^L^, 80.5^R^ | N/A |
| Depth of pubic ‘boot,’ anteroposterior | N/A | 82.4^L^, 84.7^R^ | N/A |
|  |  |  |  |
| *Ischium* |  |  |  |
| Length, proximodistal | N/A | 275^L^*, 305^R^ | N/A |
| Length to obturator process, proximodistal | N/A | 109.8^L^, 110.1^R^ | N/A |
| Length from obturator process, proximodistal | N/A | 210^L^*, 225^R^ | N/A |
|  |  |  |  |
| *Femur* |  |  |  |
| Length, proximodistal | 525^R^ | 505^L^, 500^R^ | N/A |
| Width of proximal end, mediolateral | 121.7^L^*, 127.4^R^ | 136.3^L^, 135.5^R^ | N/A |
| Width of distal end, mediolateral | 94.1^L^*, 112.4^R^ | 110.0^R^ | N/A |
|  |  |  |  |
| *Tibia* |  |  |  |
| Length, proximodistal | 660^L^ | 595^L^ | N/A |
| Width of proximal end, mediolateral | 93.5^L^, 101.7^R^* | 86.1^L^ | N/A |
| Depth of proximal end, anteroposterior | 104.6^L^ | 88.4^L^ | N/A |
| Width of distal end, mediolateral | 101.7^L^ | 110.3^L^ | N/A |
| Depth of distal end, anteroposterior | 44.0^L^ | N/A | N/A |
|  |  |  |  |
| *Fibula* |  |  |  |
| Length, proximodistal | 585^L^, 580^R^ | 570^L^ | N/A |
| Width of proximal end, anteroposterior | 70.0^L^, 65.0^R^ | 74.7^L^, 67.9^R^ | N/A |
| Width of proximal end, mediolateral | 29.2^L^, 27.5^R^ | 25.0^L^, 31.8^R^ | N/A |
|  |  |  |  |
| *Astragalocalcaneum* |  |  |  |
| Length, proximodistal | 133.1^L^*, 141.8*^R^ | 220^L^ | N/A |
| Width across distal condyles, mediolateral | 98.5^L^, 98.0^R^ | 109.7^L^ | N/A |
|  |  |  |  |
| *Metatarsal I* |  |  |  |
| Length, proximodistal | 76.0^R^ | N/A | N/A |
|  |  |  |  |
| *Metatarsal V* |  |  |  |
| Length, proximodistal | N/A | 114.4^L^ | N/A |
| Depth of proximal end, anteroposterior | 16.0^L^* | 20.7^L^ | N/A |
|  |  |  |  |
| *Pedal phalanx I-1* |  |  |  |
| Length, proximodistal | 101.1 | N/A | N/A |
|  |  |  |  |
| *Pedal phalanx III-1* |  |  |  |
| Length, proximodistal | 114.2 | N/A | N/A |
|  |  |  |  |
| *Pedal phalanx III-3* |  |  |  |
| Length, proximodistal | N/A | 85.9 | N/A |
|  |  |  |  |
| *Pedal phalanx III-4 (ungual)* |  |  |  |
| Length, proximodistal | 101.1 | N/A | N/A |
| Length along curved dorsal surface, proximodistal | 130.0 | N/A | N/A |
|  |  |  |  |
| *Pedal phalanx IV-2* |  |  |  |
| Length, proximodistal | 63.7 | N/A | N/A |
|  |  |  |  |
| *Pedal phalanx IV-3* |  |  |  |
| Length, proximodistal | 52.5 | N/A | N/A |
|  |  |  |  |
| *Pedal phalanx IV-4* |  |  |  |
| Length, proximodistal | 56.5 | N/A | N/A |

**(4) Supplementary information on phylogenetic analysis**

**Table S3. Sources of information for scoring taxa included in the phylogenetic data matrix of**  for 25 newly added and eight modified characters.

| **Taxon** | **Source(s)** |
| --- | --- |
| *Herrerasaurus ischigualastensis* |  |
| *Velociraptor mongoliensis* |  |
| *Archaeopteryx lithographica* |  |
| *Incisivosaurus gauthieri* |  |
| *Caudipteryx zoui* | ; M.C.L., pers. obs. |
| *Avimimus portentosus* |  |
| *Microvenator celer* |  |
| *Oviraptor philoceratops* |  |
| *Rinchenia mongoliensis* |  |
| *Citipati osmolskae* |  |
| Zamyn Khondt oviraptorid |  |
| *Khaan mckennai* |  |
| *Conchoraptor gracilis* |  |
| *Machairasaurus leptonychus* |  |
| *Nemegtomaia barsboldi* |  |
| *Heyuannia huangi* |  |
| *Ingenia yanshini* |  |
| *Gigantoraptor erlianensis* | ; M.C.L., pers. obs. |
| *Caenagnathasia martinsoni* |  |
| *Elmisaurus elegans* |  |
| *Leptorhynchos gaddisi* |  |
| *Chirostenotes pergracilis* |  |
| *Caenagnathus collinsi* |  |
| *Anzu wyliei* (= ‘*Caenagnathus* sp. Hell Creek’ of ) | This paper |
| *Hagryphus giganteus* |  |
| *Elmisaurus rarus* |  |
| *Nomingia gobiensis* |  |
| *Epichirostenotes curriei* |  |

**Table S4. Scores changed for *Khaan mckennai* in matrix of Longrich et al. based on information from Balanoff and Norell** .

| **Character** |  | **This paper** |
| --- | --- | --- |
| 3 | ? | 2 |
| 11 | ? | 1 |
| 28 | ? | 1 |
| 44 | ? | 1 |
| 45 | ? | 1 |
| 46 | ? | 1 |
| 52 | ? | 1 |
| 53 | ? | 1 |
| 56 | ? | 0 |
| 73 | ? | 1 |
| 74 | ? | 1 |
| 77 | ? | 1 |
| 93 | ? | 1 |
| 94 | ? | 1 |
| 101 | ? | 1 |
| 104 | 1 | ? |
| 105 | 1 | ? |
| 114 | ? | 0 |
| 116 | ? | 0 |
| 129 | ? | 0 |
| 131 | 0 | 1 |
| 139 | ? | 1 |
| 147 | ? | 2 |
| 150 | ? | 1 |
| 152 | ? | 1 |
| 154 | ? | 1 |
| 155 | ? | 1 |
| 156 | ? | 0 |
| 176 | 0 | 1 |
| 192 | 0 | 1 |

**Table S5. Specimens used for rescoring caenagnathid taxa from the Campanian of Alberta included in the phylogenetic analysis of Longrich et al. .**

| **Taxon** | **Specimen(s)** |
| --- | --- |
| *Caenagnathus collinsi* | CMN 8776 (holotype) |
| *Chirostenotes pergracilis* | CMN 2367 (holotype), TMP 1979.020.0001 |
| *Elmisaurus* *elegans* | ROM 781 (holotype), TMP 1982.039.0004, ROM 37163, TMP 1982.016.0006, TMP 1996.012.0141 |

**Table S6. Scores changed for caenagnathid taxa from the Campanian of Alberta in the phylogenetic data matrix of Longrich et al.** .

| ***Caenagnathus collinsi*** | | | |
| --- | --- | --- | --- |
| Character |  | This paper | Justification |
| 78 | ? | 1 | Reinterpretation of morphology |
| 93 | ? | 1 | Reinterpretation of morphology |
| 157 | 0 | ? | Removal of CMN 8538 |
| 158 | 1 | ? | Removal of CMN 8538 |
| 159 | 1 | ? | Removal of CMN 8538 |
| 179 | 0 | ? | Removal of CMN 8538 |
| 185 | ? | 1 | Reinterpretation of morphology |
| 186 | ? | 0 | Reinterpretation of morphology |
| 187 | ? | 1 | Reinterpretation of morphology |
| 188 | ? | 1 | Reinterpretation of morphology |
| 189 | ? | 2 | Reinterpretation of morphology |
| 190 | ? | 1 | Reinterpretation of morphology |
| 191 | ? | 0 | Reinterpretation of morphology |
| 193 | ? | 1 | Reinterpretation of morphology |
| 194 | ? | 1 | Reinterpretation of morphology |
| 195 | ? | 1 | Reinterpretation of morphology |
| 196 | ? | 1 | Reinterpretation of morphology |
| 198 | ? | 1 | Reinterpretation of morphology |
|  | | | |
| ***Chirostenotes pergracilis*** | | | |
| Character |  | This paper | Justification |
| 71 | 1 | ? | Removal of mandibular material |
| 72 | 1 | ? | Removal of mandibular material |
| 73 | 2 | ? | Removal of mandibular material |
| 74 | 1 | ? | Removal of mandibular material |
| 75 | 1 | ? | Removal of mandibular material |
| 76 | 1 | ? | Removal of mandibular material |
| 77 | 1 | ? | Removal of mandibular material |
| 78 | 1 | ? | Removal of mandibular material |
| 79 | 0 | ? | Removal of mandibular material |
| 80 | 2 | ? | Removal of mandibular material |
| 81 | 0 | ? | Removal of mandibular material |
| 82 | 1 | ? | Removal of mandibular material |
| 83 | 1 | ? | Removal of mandibular material |
| 84 | 2 | ? | Removal of mandibular material |
| 85 | 0 | ? | Removal of mandibular material |
| 86 | 0 | ? | Removal of mandibular material |
| 87 | 1 | ? | Removal of mandibular material |
| 88 | 1 | ? | Removal of mandibular material |
| 89 | 1 | ? | Removal of mandibular material |
| 90 | 1 | ? | Removal of mandibular material |
| 91 | 1 | ? | Removal of mandibular material |
| 92 | 1 | ? | Removal of mandibular material |
| 94 | 1 | ? | Removal of mandibular material |
| 95 | 1 | ? | Removal of mandibular material |
| 96 | 2 | ? | Removal of mandibular material |
| 99 | 2 | ? | Removal of mandibular material |
| 121 | 0 | 1 | Reinterpretation of morphology |
| 122 | ? | 0 | Reinterpretation of morphology |
| 133 | ? | 1 | Reinterpretation of morphology |
| 144 | ? | 1 | Reinterpretation of morphology |
| 149 | ? | 1 | Reinterpretation of morphology |
| 155 | ? | 1 | Reinterpretation of morphology |
| 164 | 0 | ? | Removal of mandibular material |
| 165 | 0 | ? | Removal of mandibular material |
| 166 | 0 | ? | Removal of mandibular material |
| 167 | 1 | ? | Removal of mandibular material |
| 168 | 0 | ? | Removal of mandibular material |
| 169 | 1 | ? | Removal of mandibular material |
| 170 | 0 | ? | Removal of mandibular material |
| 171 | 0 | ? | Removal of mandibular material |
| 184 | 1 | ? | Removal of mandibular material |
| 185 | 1 | ? | Removal of mandibular material |
| 186 | 1 | ? | Removal of mandibular material |
| 187 | 1 | ? | Removal of mandibular material |
| 188 | 1 | ? | Removal of mandibular material |
| 189 | 2 | ? | Removal of mandibular material |
| 190 | 1 | ? | Removal of mandibular material |
| 191 | 1 | ? | Removal of mandibular material |
| 192 | 1 | ? | Removal of mandibular material |
| 193 | 1 | ? | Removal of mandibular material |
| 194 | 1 | ? | Removal of mandibular material |
| 195 | 1 | ? | Removal of mandibular material |
| 196 | 1 | ? | Removal of mandibular material |
| 197 | 0 | ? | Removal of mandibular material |
| 198 | 1 | ? | Removal of mandibular material |
|  | | | |
| ***Elmisaurus elegans*** | | | |
| Character |  | This paper | Justification |
| 72 | 1 | ? | Removal of mandibular material |
| 73 | 2 | ? | Removal of mandibular material |
| 74 | 1 | ? | Removal of mandibular material |
| 75 | 1 | ? | Removal of mandibular material |
| 76 | 1 | ? | Removal of mandibular material |
| 78 | 1 | ? | Removal of mandibular material |
| 83 | 1 | ? | Removal of mandibular material |
| 84 | 2 | ? | Removal of mandibular material |
| 85 | 0 | ? | Removal of mandibular material |
| 86 | 0 | ? | Removal of mandibular material |
| 99 | 2 | ? | Removal of mandibular material |
| 164 | 0 | ? | Removal of mandibular material |
| 165 | 0 | ? | Removal of mandibular material |
| 166 | 0 | ? | Removal of mandibular material |
| 167 | 1 | ? | Removal of mandibular material |
| 171 | 0 | ? | Removal of mandibular material |
| 184 | 0 | ? | Removal of mandibular material |
| 185 | 1 | ? | Removal of mandibular material |
| 186 | 1 | ? | Removal of mandibular material |
| 187 | 1 | ? | Removal of mandibular material |
| 188 | 1 | ? | Removal of mandibular material |
| 189 | 2 | ? | Removal of mandibular material |
| 190 | 1 | ? | Removal of mandibular material |
| 192 | 1 | ? | Removal of mandibular material |
| 193 | 0 | ? | Removal of mandibular material |

**Table S7. Specimens used for scoring newly added caenagnathid OTUs from the Campanian of western Canada in phylogenetic analysis, and sources of information on each**.

| **Taxon** | **Specimen(s)** | **Source(s)** |
| --- | --- | --- |
| ‘*Caenagnathus*’ *sternbergi* | CMN 2690 (holotype), TMP 1990.056.0006, TMP 2001.012.0012 |  |
| *Macrophalangia canadensis* | CMN 8538 (holotype) |  |
| ‘Alberta dentary morph 3’ | TMP 1979.008.0622, TMP 1991.144.0001, TMP 1992.036.0390, TMP 1992.040.0044 |  |

**Table S8**. Sources of character information for ten oviraptorosaurian taxa newly added to phylogenetic data matrix.

| **Taxon** | **Source(s)** |
| --- | --- |
| *Banji long* |  |
| *Caudipteryx dongi* |  |
| *Ganzhousaurus nankangensis* |  |
| *Jiangxisaurus ganzhouensis* |  |
| *Nankangia jiangxiensis* |  |
| *Ojoraptorsaurus boerei* |  |
| *Shixinggia oblita* |  |
| *Similicaudipteryx yixianensis* |  |
| *Wulatelong gobiensis* |  |
| *Yulong mini* |  |

**Table S9. Sources for geological provenance and age of 33 taxa (31 oviraptorosaurs) shown in Figure 7 (the** calibrated phylogeny).

| **Taxon** | **Formation(s)** | **Source(s) (formation)** | **Age** | **Source(s) (age)** |
| --- | --- | --- | --- | --- |
| *Velociraptor mongoliensis* | Djadokhta |  | Campanian (early–middle) |  |
| *Archaeopteryx lithographica* | Solnhofen |  | Tithonian (early) |  |
| *Incisivosaurus gauthieri* | Yixian |  | Aptian (early) |  |
| *Similicaudipteryx yixianensis* | Jiufotang |  | Aptian (middle) |  |
| *Caudipteryx dongi* | Yixian |  | Aptian (early) |  |
| *Caudipteryx zoui* | Yixian |  | Aptian (early) |  |
| *Avimimus portentosus* | ?Barun Goyot, Nemegt |  | Campanian (middle)?–  Maastrichtian (early) |  |
| *Microvenator celer* | Cloverly |  | Albian (middle–late) |  |
| *Gigantoraptor erlianensis* | Iren Dabasu |  | Santonian |  |
| *Caenagnathasia martinsoni* | Bissekty |  | Turonian (late) |  |
| Alberta dentary morph 3 | Dinosaur Park |  | Campanian (late) |  |
| *Leptorhynchos gaddisi* | Aguja |  | Campanian (late) |  |
| ‘*Caenagnathus*’ *sternbergi* | Dinosaur Park |  | Campanian (late) |  |
| *Anzu wyliei* | Hell Creek | This paper | Maastrichtian (late) |  |
| *Caenagnathus collinsi* | Dinosaur Park |  | Campanian (late) |  |
| *Nankangia jiangxiensis* | Nanxiong |  | Campanian–Maastrichtian |  |
| *Yulong mini* | Qiupa |  | Campanian?–Maastrichtian (late) |  |
| *Nomingia gobiensis* | Nemegt |  | Maastrichtian (early) |  |
| *Oviraptor philoceratops* | Djadokhta |  | Campanian (early–middle) |  |
| *Rinchenia mongoliensis* | Nemegt |  | Maastrichtian (early) |  |
| Zamyn Khondt oviraptorid | Djadokhta |  | Campanian (early–middle) |  |
| *Citipati osmolskae* | Djadokhta |  | Campanian (early–middle) |  |
| *Wulatelong gobiensis* | Wulansuhai |  | Campanian (early–middle) |  |
| *Banji long* | Nanxiong |  | Campanian–Maastrichtian |  |
| *Shixinggia oblita* | Pingling |  | Maastrichtian |  |
| *Jiangxisaurus ganzhouensis* | Nanxiong |  | Campanian–Maastrichtian |  |
| *Ganzhousaurus nankangensis* | Nanxiong |  | Campanian–Maastrichtian |  |
| *Nemegtomaia barsboldi* | Barun Goyot, Nemegt |  | Campanian (middle)–  Maastrichtian (early) |  |
| *Machairasaurus leptonychus* | Bayan Mandahu |  | Campanian (early–middle) |  |
| *Conchoraptor gracilis* | Barun Goyot |  | Campanian (middle–late) |  |
| *Khaan mckennai* | Djadokhta |  | Campanian (early–middle) |  |
| *Ingenia yanshini* | Barun Goyot, Nemegt |  | Campanian (middle)–  Maastrichtian (early) |  |
| *Heyuannia huangi* | Dalangshan |  | Maastrichtian? |  |

­­­­

**Appendix S1. Descriptions of characters used in phylogenetic analysis.**

**A. Characters originally from and references therein. Characters modified in subsequent works are indicated with an asterisk adjacent to the character number, and the work in question is provided in parentheses.**

1*. Ratio of the preorbital skull length to the basal skull length: 0.6 or more (0); less than 0.6 (1) .

2. Pneumatized crest-like prominence on the skull roof: absent (0); present (1).

3*. Ratio of the width (across premaxilla–maxilla suture) of the snout to its length: less than 0.3 (0); 0.3–0.4 (1); more than 0.4 (2) .

4. Ratio of the length of the tomial margin of the premaxilla to the premaxilla height (ventral to the external naris): 1.0–1.4 (0); more than 1.7 (1); 0.7 or less (2).

5. Inclination of the anteroventral margin of the premaxilla relative to the horizontally positioned ventral margin of the jugal: vertical (0); posterodorsal (1); anterodorsal (2).

6. Ventral projection of the premaxilla below the ventral margin of the maxilla: absent (0); small (1); significant (2).

7. Share of the premaxilla (ventral) in the basal skull length: 0.10 or less (0); 0.12 or more (1).

8. Pneumatization of the premaxilla: absent (0); present (1).

9*. Ratio of the length of the maxilla (in lateral view) to the basal skull length: 0.4–0.7 (0); less than 0.4 (1) .

10. Subantorbital portion of the maxilla: not inset medially (0); inset medially (1).

11. Palatal shelf of the maxilla with two longitudinal ridges and a tooth-like ventral process: absent (0); present (1).

12.Ventral margins of maxilla and jugal: margins form a straight line (0); the ventral margin of the maxilla slopes anteroventrally, its longitudinal axis at an angle of ca. 120° to the longitudinal axis of the jugal (1).

13. Rim around antorbital fossa: well pronounced (0); poorly delimited (1).

14. Antorbital fossa: bordered anteriorly by the maxilla (0); bordered anteriorly by the premaxilla (1).

15. Accessory maxillary fenestrae: absent (0); at least one accessory fenestra present (1).

16. Nasal along midline: longer than frontal (0); shorter than or as long as the frontal (1).

17.Nasals: separate (0); fused (1).

18. Subnarial process of the nasal: long (0); short (1).

19. Shape of the narial opening: longitudinally oval (0); teardrop-shaped, slightly longer than wide (1); much longer than wide (2).

20. Nasal recesses: absent (0); present (1).

21. External naris position relative to the antorbital fossa: naris and fossa widely separated (0); posterior margin of the naris reaching the fossa (1); overlapping anterodorsally most of the fossa (2).

22. Ventral margin of the external naris: at the level of the maxilla (0); dorsal to the maxilla (1).

23. Prefrontal: present (0); absent or fused with the lacrimal (1).

24. Lacrimal shaft: not projecting outward beyond the orbital plane and lateral surface of the snout (0); the middle part of the shaft projecting laterally to form a flattened transverse bar in front of the eye (1).

25. Lacrimal recesses: absent (0); present (1).

26. Ratio of the length of the orbit to the length of the antorbital fossa: 0.7–0.9 (0); 1.2 or more (1).

27. Ratio of the length of the parietal to the length of the frontal: 0.6 or less (0); 1.0 or more (1).

28. Pneumatization of skull roof bones: absent (0); present (1).

29. Sagittal crest along the interparietal contact: absent (0); present (1).

30. Supratemporal fossa: invading the frontal (0); not invading the frontal (1).

31. Infratemporal fenestra: dorsoventrally elongate, narrow anteroposteriorly (0); subquadrate, its anteroposterior length comparable to the orbital length (1).

32. Pneumatization of the squamosal: absent (0); present (1).

33.Cotyle-like incision on the ventrolateral margin of the squamosal (for reception of the dorsal end of the ascending process of the quadratojugal): absent (0); present (1).

34. Ventral ramus of the jugal: deep dorsoventrally and flattened mediolaterally (0); shallow dorsoventrally or rod‑shaped (1).

35.Jugal process of the postorbital: not extending ventrally below two-thirds of the orbit height (0); long, extending ventrally close to the base of the postorbital process of the jugal (1).

36.Postorbital process of the jugal: posterodorsally inclined (0); perpendicular to the ventral ramus of the jugal (1); absent (2).

37. Jugal–postorbital contact: present (0); absent (1).

38. Quadratojugal process of the jugal in lateral view: forked (0); not forked (1); fused with the quadratojugal (2).

39. Quadratojugal–squamosal contact: absent (0); present (1).

40. Ascending (squamosal) process of the quadratojugal: bordering ca. the ventral half, or less, of the infratemporal fenestra (0); bordering the ventral two-thirds or more of the infratemporal fenestra (1); absent (2).

41.Angle between the ascending and jugal processes of the quadratojugal: ca. 90° (0); less than 90° (1).

42.Quadrate process of the quadratojugal: well developed, extending posteriorly or posteroventrally beyond the posterior margin of the ascending process (0); not extending beyond the posterior margin of the ascending process (1).

43. Dorsal part of the quadrate: erect (0); bent backward (1).

44. Otic process of the quadrate: articulating only with the squamosal (0); articulating with the squamosal and the lateral wall of the braincase (1).

45. Pneumatization of the quadrate: absent (0); present (1).

46. Lateral accessory process on the distal end of the quadrate for articulation with the quadratojugal: absent (0); present (1).

47. Lateral cotyle for the quadratojugal on the quadrate: absent (0); present (1).

48. Mandibular condyles of quadrate: posterior to the occipital condyle (0); in the same vertical plane as the occipital condyle (1); anterior to the occipital condyle (2).

49. Nuchal transverse crest: pronounced (0); not pronounced (1).

50.Occiput position in relation to the ventral margin of the jugal–quadratojugal bar: about perpendicular (0); inclined anterodorsally (1).

51. Paroccipital process: directed laterally (0); directed ventrally (1).

52. Foramen magnum: smaller than or equal in size to the occipital condyle (0); larger than the occipital condyle (1).

53. Basal tubera: modestly pronounced (0); well developed, widely separated (1).

54. Pneumatization of the basisphenoid: weak or absent (0); extensive (1).

55. Basipterygoid processes: well developed (0); strongly reduced (1); absent (2).

56. Parasphenoid rostrum: horizontal or anterodorsally directed (0); sloping anteroventrally (1).

57. Depression in the periotic region: absent (0); present (1).

58. Pneumatization of the periotic region: absent or weak (0); extensive (1).

59. Quadrate ramus of the pterygoid: distant from the braincase wall (0); overlapping the braincase (1).

60. Pterygoid basal process for contact with the basisphenoid: absent (0); present (1).

61. Ectopterygoid position: lateral to the pterygoid (0); anterior to the pterygoid (1).

62. Ectopterygoid contacts with the maxilla and lacrimal: absent (0); present (1).

63. Ectopterygoid: short anteroposteriorly with a hook-like jugal process (0); elongate, shaped like a Viking ship, without a hook-like process (1).

64. Massive pterygoid–ectopterygoid longitudinal bar: absent (0); present (1).

65. Palate extending below the cheek margin: absent (0); present (1).

66. Palatine: tetraradiate or trapezoidal (0); triradiate, without a jugal process (1); developed in horizontal, longitudinal, and transverse planes perpendicular to each other (2).

67. Pterygoid wing of the palatine: dorsal to the pterygoid (0); ventral to the pterygoid (1).

68. Maxillary process of the palatine: shorter than the vomeral process (0); longer than the vomeral process (1).

69. Vomer: distant from the parasphenoid rostrum (0); approaching or in contact with the parasphenoid rostrum (1).

70. Suborbital (ectopterygoid–palatine) fenestra: well developed (0); closed or reduced (1).

71. Jaw joint: distant from the midline of the skull (0); close to the skull midline (1).

72. Movable intramandibular joint: present (0); suppressed (1).

73. Mandibular symphysis: loose (0); tightly sutured (1); fused (2).

74. Extended symphyseal shelf at the mandibular symphysis: absent (0); present (1).

75. Downturned symphyseal portion of the dentary: absent (0); present (1).

76. U‑shaped mandibular symphysis: absent (0); present (1).

77. Ratio of the length of the retroarticular process to the total mandibular length: less than 0.05 or the process absent (0); ca. 0.10 (1).

78*. Dentary: elongate (0); proportionately short and deep, with maximum depth of dentary between 25% and 50% of dentary length (with length measured from the tip of the jaw to the end of the posterodorsal process) (1); extremely short and deep, with maximum depth 50% or more of dentary length (2) (ORDERED) .

79. Ratio of the height of the external mandibular fenestra to the length of the fenestra: 0.2–0.5 (0); 0.7–1.0 (1); fenestra absent (2).

80. Ratio of the length of the external mandibular fenestra to total mandibular length: 0.15–0.20 (0); not more than 0.10 or fenestra absent (1); 0.25 or more (2).

81*. Process of the surangular dividing the external mandibular fenestra: absent (0); short and broad (1); elongate and spike-like (2) (ORDERED) .

82. Coossification of the articular with the surangular: absent (0); present (1).

83. Mandibular rami in dorsal view: straight (0); laterally bowed at midlength (1).

84*. Anterodorsal margin of dentary in lateral view: straight (0); concave (1); broadly concave (2) (ORDERED) .

85. Posterior margin of the dentary: incised, producing two posterior processes (0); oblique (1).

86. Posterodorsal process of the dentary long and shallow: present (0); absent (1).

87. Posteroventral process of the dentary shallow and long, extending posteriorly at least to the posterior border of the external mandibular fenestra: absent (0); present (1).

88*. Coronoid process: posteriorly positioned and vertically projected (0); anteriorly positioned, near the midpoint of the jaw, with a medially hooked apex (1) .

89. Surangular foramen: present (0); absent (1).

90. Mandibular articular facet for the quadrate: comprising the surangular and the articular (0); formed exclusively of the articular (1).

91. Mandibular articular facet for the quadrate: with one or two cotyles (0); convex in lateral view, transversely wide (1).

92*. Position of the quadrate articular surface relative to the level of the adjoining dorsal margin of the mandibular ramus: ventral (0); moderately elevated, quadrate articulation grades smoothly into remainder of mandible (1); highly elevated, anterior and posterior margins of quadrate articulation at nearly right angles to remainder of mandible (2) (ORDERED) (this paper). In the mandibles of most theropods, the quadrate articulation is concave in lateral view, and as such, the quadrate articular surface is situated ventral to the adjoining dorsal margin of the mandibular ramus. In most oviraptorosaurs, however—with the exceptions of *Avimimus portentosus* and possibly *Caudipteryx zoui*—the quadrate articulation is convex in lateral view, with its anterior and posterior margins grading smoothly into the remainder of the mandible. As a result, the quadrate articular surface is elevated above the adjoining region of the mandibular ramus. This condition is taken to an extreme in *Gigantoraptor erlianensis* and ‘*Caenagnathus*’ *sternbergi*. In these taxa, the anterior and posterior margins of the quadrate articulation meet the remainder of the mandible at nearly right angles, and the quadrate articular surface is elevated well above the rest of the posterior half of the mandible. (Replaces 92. Position of the articular facet for the quadrate: ventral to the level of the adjoining dorsal margin of the mandibular ramus (0), dorsal to this margin (1).)

93. Anterior part of the prearticular: deep, approaching the dorsal margin of the mandible (0); shallow, strap-like, not approaching the dorsal mandibular margin (1).

94. Splenial: subtriangular, approaching the dorsal mandibular margin (0); strap-like, shallow, not approaching the margin (1).

95. Mandibular adductor fossa: anteriorly delimited, occupying the posterior part of the mandible (0); large, anteriorly and dorsally extended, not delimited anteriorly (1).

96. Coronoid bone: well developed (0); strongly reduced (1); absent (2).

97. Premaxillary teeth: present (0); absent (1).

98. Maxillary tooth row: extends at least to the level of the preorbital bar (0); does not reach the level of the preorbital bar (1); maxillary teeth absent (2).

99*. Dentary teeth: present (0); absent from tip of jaw but present posteriorly (1); absent (2) (ORDERED) .

100. Number of cervicals (excluding cervicodorsal): not more than 10 (0); more than 10 (1).

101. Anterior articular facets of the centra in the anterior postaxial cervicals: not inclined or only slightly inclined (0); strongly inclined posteroventrally, almost continuous with the ventral surfaces of the centra (1).

102. Centra of the anterior cervicals: not extending posteriorly beyond their respective neural arches (0); extending posteriorly beyond their respective neural arches (1).

103. Epipophyses on the postaxial cervicals: in the form of a low crest or rugosity (0); prong‑shaped (1).

104. Cervical ribs in adults: loosely attached to vertebrae (0); firmly attached (1); fused (2).

105. Shafts of cervical ribs: longer than their respective centra (0); not longer than their respective centra (1).

106. Lateral pneumatic fossae (‘pleurocoels’) on the dorsal centra: absent (0); present (1).

107.Ossified uncinate processes on the dorsal ribs: absent (0); present (1).

108. Number of vertebrae included in the synsacrum in adults: not more than 5 (0); 6 (1); 7–8 (2).

109. Sacral spines in adults: unfused (0); fused (1).

110. Lateral pneumatic fossae on the sacral centra: absent (0); present (1).

111. Transition point on the caudals: absent (0); present (1).

112. Number of caudals with transverse processes: 15 or more (0); fewer than 15 (1).

113. Lateral pneumatic fossae on the caudal centra: absent (0); present at least in the anterior part of the tail (1).

114. Neural spines confined to: at least 23 anterior caudals (0); at most 16 anterior caudals (1).

115. Number of caudals: more than 35 (0); 30 or fewer (1).

116. Posterior caudal prezygapophyses: overlapping less than half of the centrum of the preceding vertebra (0); overlapping at least half of the centrum of the preceding vertebra (1).

117. Hypapophyses in the cervicodorsal vertebral region: absent (0); small (1); prominent (2).

118. Posterior hemal arches: deeper than long (0); longer than deep (1).

119. Ratio of the length of the scapula to the length of the humerus: 0.8–1.1 (0); 1.2 or more (1); 0.7 or less (2).

120. Acromion: projecting dorsally (0); projecting anteriorly (1); everted laterally (2).

121. Posteroventral process of the coracoid: absent or short, not extending beyond the glenoid diameter (0); long, posteroventrally extending beyond the glenoid (1).

122. Orientation of the glenoid on the pectoral girdle: posteroventral (0); lateral (1).

123. Deltopectoral crest: low, its width equal to, or smaller than, the shaft diameter (0); expanded, wider than the shaft diameter (1).

124. Extent of the deltopectoral crest (measured from the humeral head to the apex of the crest): about the proximal third of the humerus length or less (0); ca. 40%–50% of the humerus length (1).

125. Shaft of the ulna: straight (0); bowed, convex posteriorly (1).

126. Ratio of the length of the radius to the length of the humerus: 0.80 or less (0); 0.85 or more (1).

127. Combined lengths of manual phalanges III‑1 and III‑2: greater than the length of phalanx III‑3 (0); less than or equal to the length of phalanx III‑3 (1).

128. Ratio of the length of metacarpal I to the length of metacarpal II: 0.5 or more (0); less than 0.5 (1).

129. Proximal margin of metacarpal I in dorsal view: straight, horizontal (0); angled due to a medial extent of the carpal trochlea (1).

130. Metacarpal II relative to metacarpal III: shorter (0); longer (1); subequal (2).

131. Ratio of the length of metacarpal II to the length of the humerus: 0.4 or less (0); more than 0.4 (1).

132. Ratio of the length of the manus to the length of the humerus plus the radius: 0.50–0.65 (0); more than 0.65 (1); less than 0.50 (2).

133. Ratio of the length of the manus to the length of the femur: 0.3–0.6 (0); more than 0.7 (1).

134*. Ratio of the length of the humerus to the length of the femur: 0.50–0.69 (0); 0.70 or more (1) .

135. Dorsal margins of opposite iliac blades: well separated from each other (0); close to or contacting each other along their medial sections (1).

136. Dorsal margin of the ilium along the central portion of the blade: straight (0); arched (1).

137. Preacetabular process of the ilium relative to the postacetabular process (lengths measured from the center of the acetabulum): shorter or equal (0); longer (1).

138. Preacetabular process: not expanded or weakly expanded ventrally below the level of the dorsal acetabular margin (0); expanded ventrally well below the level of the dorsal acetabular margin (1).

139. Morphology of the ventral margin of the preacetabular process: cuppedicus fossa absent, margin transversely narrow (0); cuppedicus fossa or a wide shelf present (1); margin flat, wide at least close to the pubic peduncle (2).

140. Anteroventral extension of the preacetabular process: absent (0); with rounded tip (1); hook-like (2).

141. Posterior end of the postacetabular process: truncated or broadly rounded (0); narrowed or acuminate (1).

142. Anteroposterior length of the pubic peduncle: about the same as that of the ischial peduncle (0); distinctly greater than that of the ischial peduncle (1).

143. Dorsoventral extension of the pubic peduncle: level with the ischial peduncle (0); deeper than the ischial peduncle (1).

144*. Ratio of the length of the ilium to the length of the femur: 0.50–0.79 (0); 0.80 or more (1) .

145. Pelvis: propubic (0); mesopubic (1); opisthopubic (2).

146. Pubic shaft: straight (0); concave anteriorly (1).

147. Pubic foot: anterior and posterior processes about equally long (0); anterior process absent or shorter than the posterior process (1); anterior process longer than the posterior process (2).

148. Posterior margin of the ischial shaft: straight or almost straight (0); distinctly concave (1).

149. Greater trochanter of the femur: weakly separated, or not separated, from the femoral head (0); distinctly separated from the femoral head (1).

150. Anterior and greater trochanters: separated (0); contacting (1).

151. Dorsal extremity of the anterior trochanter: well below the greater trochanter (0); about level with the greater trochanter (1).

152. Fourth trochanter: well developed (0); weakly developed or absent (1).

153. Adductor fossa and the associated anteromedial crest on the distal femur: weak or absent (0); well developed (1).

154. Distal projection of the fibular condyle of the femur beyond the tibial condyle: absent (0); present (1).

155. Ascending process of the astragalus: as tall as wide across the base (0); taller than wide (1).

156. Distal tarsals: not fused with the metatarsus (0); fused with the metatarsus (1).

157. Proximal coossification of metatarsals II–IV: absent (0); present (1).

158*. Arctometatarsus: absent (0); present, but only proximalmost extreme of metatarsal III obscured from anterior view in articulated metatarsus (1); present, proximal ~half of metatarsal III obscured from anterior view in articulated metatarsus (2) (this paper). Many oviraptorosaurs lack an arctometatarsus. In articulated metatarsi of caenagnathids, however, the proximal extreme of metatarsal III is ‘pinched’ between metatarsals II and IV and consequently obscured from anterior view. In the coossified tarsometatarsus of the highly aberrant *Avimimus* *portentosus*, the proximal ~half of metatarsal III is obscured from anterior view. (Replaces 158. Arctometatarsus: absent (0), present (1).)

159. Length of metatarsal I constituting: more than 50% of metatarsal II length (0); less than 50% of metatarsal II length (1); metatarsal I absent (2).

160. Ratio of the maximum length of the metatarsus to the length of the femur: 0.4–0.6 (0); ca. 0.3 (1); 0.7–0.8 (2).

161. Crenulated tomial margin of the premaxilla: absent (0); present (1).

**B. Characters originally from . Characters modified in subsequent works are indicated with an asterisk adjacent to the character number, and the work in question is provided in parentheses.**

162. Frontals: flat or weakly arched, not strongly projecting above orbit in lateral view (0); strongly arched, projecting well above orbit in lateral view to contribute to nasal–frontal crest (1).

163. Exoccipital: short, weakly projecting (0); strongly projects ventrally beyond squamosal in lateral view, approaching ventral end of quadrate (1).

164. Dentary posterodorsal ramus: straight or weakly curved (0); strongly bowed dorsally (1).

165. Dentary symphyseal ventral process: absent (0); prominent process present on posteroventral surface of symphysis (1).

166. Dentary anteroventral margin in lateral view: straight or weakly downturned (0); strongly downturned (1).

167. Lateral surface of dentary: smooth (0); bearing a deep fossa, sometimes with associated pneumatopore (1).

168. Angular: contributes extensively to the border of the external mandibular fenestra (0); largely excluded by surangular (1).

169. Surangular with an anteroposteriorly elongate flange on the ventral edge: absent (0); present (1).

170. External mandibular fenestra: elongate (0); height subequal to length (1).

171. Dentary contribution to external mandibular fenestra relative to length of dentary: no more than 50% (0); exceeds 50% (1).

172. Metacarpal I expanded ventrally to cover ventral surface of metacarpal II: absent (0); present (1).

173. Unguals of manual digits II and III: strongly curved (0); weakly curved (1).

174. Manual phalanx I-1: slender (0); more robust than II-1 (1); more than 200% diameter of II-1 (2) (ORDERED). phrased this character as “Manus I-1 slender (0) or more robust than II (1) or more than 200 per cent diameter of II-1 (2).” We assume that state 1 is “more robust than II-1.”

175. Manual phalanx III-3: longer than phalanx III-2 (0); shorter than or equal in length to III-2 (1).

176*. Manual phalanx II-2: longer than II-1 (0); subequal to or slightly shorter than II-1 (1); distinctly shorter than II-1 (2) (ORDERED) .

177*. Manual digit II: elongate, with combined lengths of manual phalanges II-1 and II-2 longer than metacarpal II (0); combined lengths of manual phalanges II-1 and II-2 subequal to metacarpal II (1) (this paper). Replaces character 177 of : “Manual digit II elongate, with combined lengths of manual phalanges II-1 and II-2 subequal to or longer than metacarpal II (0), or combined lengths of manual phalanges II-1 and II-2 subequal to metacarpal II (1), or combined lengths of manual phalanges II-1 and II-2 shorter than metacarpal II (2) (ORDERED).”

178. Ischium strongly bent posteriorly at midshaft, distal end forms an angle of at least 60º with proximal end: absent (0); present (1).

179. Metatarsus: elongate (0); short, length does not exceed 300% of proximal width (1).

180. Ilium: tall (0); low and anteroposteriorly elongate, height less than 25% of length (1).

181. Anterior blade of ilium shallower than posterior blade: absent (0); present (1).

**C. Characters from and references therein. See for detailed character descriptions.**

182. External naris: placed anteriorly (0); extends posteriorly, with posterior end lying above antorbital fenestra (1).

183. Premaxillae, nasal processes anteroposteriorly expanded and mediolaterally compressed to form a bladelike internarial bar: absent (0); present (1).

184. Dentary, anterodorsal tip of beak: projecting upwards (0); projecting anterodorsally, tip of beak projecting at an angle of 45° or less relative to the ventral margin of the symphysis (1).

185. Dentary symphysis with interior surface bearing vascular grooves and associated foramina: absent (0); present (1).

186. Dentary symphysis bearing an hourglass-shaped ventral depression: absent (0); present (1).

187. Meckelian groove terminates: on the inside of the dentary (0); on the ventral surface of the symphysis (1).

188. Lingual triturating shelf: absent (0); present (1).

189. Symphyseal ridges inside the tip of the beak: absent (0); present but weakly developed (1); present and well developed (2) (ORDERED).

190. Lingual ridges inside the lateral occlusal surface of beak: absent (0); present (1).

191. Posteroventral process of dentary: straight (0); bowed ventrally (1).

192. Dentaries pneumatized: absent (0); present (1).

193. Dentary: participates in dorsal border of the external mandibular fenestra (0); excluded by anterior extension of the surangular (1).

194. Dentary: participates in ventral border of external mandibular fenestra (0); excluded by anterior extension of the angular (1).

195. Surangular and angular divided by posterior extension of the external mandibular fenestra: absent (0); present (1).

196. Posterior end of the surangular: deep (0); shallow, subequal to or shallower than angular (1).

197. Surangular: deep anteriorly (0); strap-like (1).

198. Retroarticular process extends: posteriorly (0); posteroventrally (1).

199. Metacarpal I: proportionately broad (0); long and slender, diameter 20% of length (1).

200. Manual phalanx I-1: longer than II-2 (0); subequal to II-2 (1); shorter than II-2 (2) (ORDERED).

201. Ischiadic peduncle of pubis with prominent medial fossa: absent (0); present (1).

202. Ischium, obturator process located: distally (0); at midshaft of ischium (1).

203. Anterior margin of obturator process: straight or convex (0); distinctly concave (1).

204. Accessory trochanter of femur: weakly developed (0); prominent, subrectangular flange or finger-like process (1).

205. Metatarsal III: with an ovoid or subtriangular cross section (0); anteroposteriorly flattened, with a concave posterior surface (1).

**D. Characters from and references therein. Character numbers in are provided in parentheses.**

206. Paroccipital process: elongate and slender, with dorsal and ventral edges nearly parallel (0); short and deep with convex distal end (1) (character 56).

207. Mandibular articulation surface: as long as ventral end of quadrate (0); twice or more as long as quadrate surface, allowing anteroposterior movement of mandible (1) (character 79).

208. Scars for interspinous ligaments in dorsal vertebrae terminate: at apex of neural spine (0); ventral to apex of neural spine (1) (character 109).

209. Sternum, distinct lateral xiphoid process posterior to costal margin: absent (0); present (1) (character 129).

210. Anterior edge of sternum: grooved for reception of coracoids (0); without grooves (1) (character 130).

211. Deltopectoral crest: large and distinct, proximal end of humerus quadrangular in anterior view (0); less pronounced, forming an arc rather than being quadrangular (1) (character 140).

212. Ischium: more than two-thirds of pubis length (0); two-thirds or less of pubis length (1) (character 173).

213. Lateral ridge of femur: absent or represented only by faint rugosity (0); distinctly raised from shaft, mound-like (1) (character 186).

**E. Characters from . Character numbers in are provided in parentheses.**

214. Surangular, distinct groove on dorsal surface: present (0); absent (1) (character 162).

215. Vomer, position: level with other palatal elements (0); ventral to other palatal elements (1) (character 163).

**F. Character from . Character number in is provided in parentheses.**

216. Calcaneum: excludes astragalus from reaching lateral margin of tarsus (0); small process of astragalus protrudes through a circular opening in edge of calcaneum to reach lateral margin of tarsus (1) (character 182).

**G. Characters from . Character numbers in are provided in parentheses.**

217. Depression on lateral surface of dentary immediately anterior to external mandibular fenestra: absent (0); present (1) (character 182).

218. Groove on ventrolateral edge of angular to receive posteroventral branch of dentary: absent (0); present (1) (character 184).

219. Posteroventral branch of dentary twisted so that lateral surface of branch faces somewhat ventrally: absent (0); present (1) (character 185).

**H. Newly formulated characters.**

220. Premaxilla, large, presumably pneumatic foramen at anteroventral corner of narial fossa: absent (0); present (1). In *Anzu wyliei*, *Khaan mckennai*, *Yulong mini*, and probably *Incisivosaurus gauthieri*, there is a large, presumably pneumatic foramen positioned at the anteroventral corner of the narial fossa. This structure was clearly illustrated in *Yulong* by Lü et al. : fig. 2A). This foramen appears to be absent (or at least much smaller) in most other oviraptorosaurs for which the premaxilla is known (e.g., *Caudipteryx zoui*, *Citipati osmolskae*, *Conchoraptor gracilis*, the Zamyn Khondt oviraptorid).

221. Accessory opening at anterodorsal extreme of snout: absent (0); present (1). As noted by Clark et al. , *Citipati osmolskae* and the unnamed Zamyn Khondt oviraptorid both possess a large, elongate accessory opening at the anterodorsal extreme of the snout. This opening is absent in other oviraptorosaurs for which this part of the skull is preserved (e.g., *Anzu wyliei*, *Banji long*, *Caudipteryx zoui*, *Conchoraptor gracilis*, *Incisivosaurus gauthieri*, *Khaan mckennai*, *Nemegtomaia barsboldi*, *Rinchenia mongoliensis*, *Yulong mini*).

222. Development of symphyseal shelf of mandible: limited, anteroposterior length of mandibular symphysis (as measured on midline) less than 20% total anteroposterior length of mandible (0); intermediate, length of symphysis greater than 20% but less than 25% length of mandible (1); extensive, length of symphysis greater than 25% mandibular length (2) (ORDERED). A symphyseal shelf is present on the mandibles of nearly all oviraptorosaurs, including basal forms such as *Caudipteryx zoui* and *Incisivosaurus gauthieri* (see character 74). Nevertheless, as noted by previous authors the degree of development of this structure varies substantially between taxa. In basal oviraptorosaurs and oviraptorids for which this condition can be evaluated (e.g., *Caudipteryx*, *Citipati osmolskae*, *Conchoraptor gracilis*, *Khaan mckennai*), the mandibular symphysis (as measured along its sagittal midline) is less than 20% the total length of the mandible (and usually much shorter than this). Conversely, in the caenagnathids *Anzu wyliei* and *Caenagnathus collinsi*, the symphysis is proportionally much longer (more than 25% the length of the mandible) due to the extreme development of the symphyseal shelf. *Gigantoraptor erlianensis* exhibits an intermediate condition. (Note that the mandible of *Anzu* is incomplete anteriorly; nevertheless, even conservative estimates of the length of its missing portion yield symphyseal lengths of greater than 25% total mandibular length. The actual symphyseal length of this taxon was probably nearly 30% mandibular length, as in *C*. *collinsi*.)

223. Prominent flange or shelf arising from lateral surface of dentary: absent (0); present (1). In both *Anzu wyliei* and *Gigantoraptor erlianensis*, a prominent shelf or flange of bone arises from the lateral surface of the dentary. The specific position and appearance of this structure differs between the two taxa, however; consequently, the respective shelf morphologies present in *Gigantoraptor* and *Anzu* are considered autapomorphic of each of these forms. Comparable bony shelves or flanges are absent from the dentaries of other oviraptorosaurs.

224. Base of retroarticular process: considerably wider mediolaterally than tall dorsoventrally (0); approximately as wide as tall (1); considerably taller than wide (2). In some oviraptorosaurs (e.g., *Avimimus portentosus*), the base (i.e., the anterior extreme, immediately posterior to the quadrate articulation) of the retroarticular process is substantially wider mediolaterally than it is tall dorsoventrally. In several others (e.g., *Caenagnathus collinsi*, *Nemegtomaia barsboldi*), these dimensions are subequal, but in *Anzu* *wyliei* and *Gigantoraptor* *erlianensis*, the base of the retroarticular process is considerably taller than wide. Xu et al. proposed this latter morphology (“retroarticular process much deeper than wide”) as a diagnostic character of *Gigantoraptor*.

225. Posteriormost caudal vertebrae fused, forming a pygostyle-like structure: absent (0); present (1). As first noted by Barsbold et al. ) and recently confirmed by Persons et al. , the caudal sequences of several oviraptorosaurs (*Citipati osmolskae*, *Conchoraptor gracilis*, *Nomingia gobiensis*, and *Similicaudipteryx yixianensis*) terminate in a series of coossified vertebrae that closely resembles the avian pygostyle. The posteriormost caudals remain unfused in several other oviraptorosaurs (*Anzu wyliei*, *Caudipteryx zoui*, *Heyuannia huangi*, *Ingenia yanshini*, and *Khaan mckennai*) and other non-avian dinosaurs.

226. Humeral shaft: straight or nearly straight (0); strongly bowed laterally (1). In most non-avian dinosaurs, including most oviraptorosaurs, the shaft of the humerus is straight or nearly straight. In *Anzu* *wyliei* and *Gigantoraptor* *erlianensis*, however, the humeral shaft is strongly bowed laterally. This morphology is most easily observed when the humerus is examined in anterior or posterior view. A laterally bowed humerus has been proposed as a diagnostic feature of *Gigantoraptor* . Given, however, that this condition is also present (albeit less marked) in *Anzu*, and that this bone remains unknown in other definitive caenagnathids, this morphology may eventually be shown to characterize this clade as a whole, or at least a broad subset of taxa within it.

227. Proximodorsal extensor ‘lip’ on manual unguals: weak (i.e., continuous or nearly continuous with remainder of dorsal surface of ungual) and/or absent (0); prominent (‘set off’ from remainder of dorsal surface by distinct change in slope immediately distal to ‘lip’) (1). In nearly all non-avian dinosaurs, including most oviraptorosaurs, the manual unguals either possess a weak proximodorsal ‘lip’ (i.e., extensor process) or lack this structure altogether. (In the manus of a few oviraptorosaurs, both of these conditions are present – for instance, in *Khaan mckennai*, the ‘lip’ is absent from manual ungual I but present in manual unguals II and III ].) In the caenagnathids *Anzu wyliei*, *Chirostenotes pergracilis*, *Elmisaurus rarus*, and *Hagryphus giganteus*, conversely, the proximodorsal ‘lip’ is extraordinarily well-developed and projected dorsally, generating a distinct change in slope along the proximodorsal margin of the ungual. *Machairasaurus leptonychus* is the only other oviraptorosaur that approaches this condition.

228. Pubic process of ischium, ‘hooked’ anterodistal extension: absent (0); present (1). In a few oviraptorosaurs (e.g., *Anzu wyliei*, *Epichirostenotes curriei*, *Nomingia gobiensis*), the pubic process of the ischium has a small but distinct anterodistal extension. This extension renders the process ‘hook-like’ in lateral view and increases the proximodistal length of its contact with the pubis.

229. Distal margin of obturator process: straight (0); distinctly concave, apex of obturator process angled distally (1). In most non-avian dinosaurs, including most oviraptorosaurs, the distal margin of the obturator process is straight. Nevertheless, in *Ingenia yanshini*, some specimens of *Khaan mckennai* , and especially *Heyuannia huangi*, the apex of the obturator process angles distally, rendering the distal margin of the process concave. The condition in *Archaeopteryx lithographica* is ambiguous due to the uncertain homologies of the ischial processes in that taxon .

230. Proximolateral edge of metatarsal IV attenuated into pointed process: absent (0); present (1). In *Elmisaurus rarus*, *Elmisaurus elegans*, and *Leptorhynchos* *gaddisi*, the proximolateral edge of metatarsal IV projects laterally, forming a distinct process that is pointed in anterior view. Other oviraptorosaurs lack this process.

**Appendix S2. Phylogenetic data matrix.** Annotated .nex and .tnt files are available from the senior author (M.C.L.) upon request.

*Herrerasaurus ischigualastensis* 0000000000 0000000000 0000000000 0000000000 0000000000 0000000000 0000000000 0000000000 0000000000 0000000000 0010?00000 00000100?0 000101??00 0000000000 0000100000 0000000000 00000?000? 0000110010 100??000?? 0000101??0 0??0000??? 00?1000?00 0000?00000

*Velociraptor mongoliensis* 0001000000 0010100101 0010100010 0000000010 0000000100 0010000000 0000000000 0000000001 0000110000 0000000100 1011011110 1101011101 -110101111 0111000011 1111201001 1100010011 000000000? 0000000011 000?0000?? 0010000100 0000000110 011?000?00 0000000000

*Archaeopteryx lithographica* 000?100000 0010110100 1100010001 ?10002110? 0?1??0020? ???00????0 000001?1?? ?100001021 000011-010 0000020100 ??0101?00? 1101100121 1100111112 1011011010 111020101? 0100?11012 00?0000?0? 0?00000001 000?0000?? 001?000000 ?010010?0? 111??00?00 000?0000?0

*Incisivosaurus gauthieri* 1001101010 00011?0111 210011?01? 00?0000?10 010?0??200 111?1?10?0 1??111?010 1?21011102 0??00011?? 11?111011? ?????????? ?????????? ?????????? ?????????? ?????????? ?????????? 0000000010 0????????? ?001000000 00000000?? ?????1???? ???00???01 000???????

*Caudipteryx zoui* 10?110?01? ?0?1110?0? 21?01?10?? ?011000111 0100?????? ?????????? ??0??????? ?0??1111?2 ???0001??? ???11?0220 ?????010?? ??0?1??10? ??1011?102 0100?00122 0110?02110 0???100012 1??0???0?? 0?00?00100 0?000?0000 0000?01000 ?0010???01 01????0?00 000?000000

*Avimimus portentosus* ?????????? 0????????? ??????1011 0??1?21212 01??00?101 111?1????? ?????????? 0?1??111?? 01?????110 00???21?21 000110?110 ??0???2?02 100111?1?? ???00000?2 10?1002010 1001111222 100010000? 0???????00 ??00?00??? ?0??0000?? 0??10110?? 11110????? ???0?0?000

*Microvenator celer* ?????????? ?????????? ?????????? ?????????? ?????????? ?????????? ?????????? ??1111?2?? ??11001??? ????????2? ??0??1???? ?????0???? 1?1011??1? ???0???112 ?11?112??? 11101????? ???0101??? 001{01}?????0 0??0000000 ?0??????0? 1??1???0?? 1?0???1??? ??0??00???

*Oviraptor philoceratops* 1????????? ?1?11????? 21???????? 1??1000??? 0????????? ?????????? ????1????? ????1?1212 2??10011?? ?1??1??22? ??0??1???? ????????1? ??101101?2 11???????? ?????????? ?????????? ?0???00100 1000000??0 ??1??????? ?????????0 ?????????? 0??11?0?0? ??0??00???

*Rinchenia mongoliensis* 11122?0111 110111???1 211111111? 1111010111 00111111?1 111121?111 1111121111 1111111212 ?011001111 1111121221 110011?101 0010102?12 101000???? ???0110112 1101????11 1111?????? 1101?00101 1????????0 0111?????? 00000001?? ??????1?10 ???1110?0? 0?0???????

*Citipati osmolskae* 11?2221?11 11011?1111 2111111?11 1?11110111 11??????11 1????11?1? 11111????1 ?11?111212 2011001111 111111122? ??????1??? ?????????? ???0?101?2 ?????????? ?????????? ?????????? 1101100101 1?01010?0? ?110000000 0000000100 ?????01010 0?01110?00 1000100?0?

Zamyn Khondt oviraptorid 1122221111 1101111111 2111111111 1111110111 1111111111 1111211111 1111121111 1111111212 2011001111 1111121221 110111?101 0010102102 1010110112 1110100121 1001112111 1111100010 1101?00101 1?0000010? ?110?00000 00000?0100 ???00?1?10 0??11???00 1?0??00??0

*Khaan mckennai* 1022111?11 11011?1121 2111111111 1?11000111 00?111?111 111??0??1? ????1????? ?111111212 1011001111 1111??1221 110??1???? 00?0102??2 10??110102 1110?00011 0001?12111 11?1100010 1001100100 1001010011 1110?000?0 0100000100 0000?01?11 00?1?10101 000?0000{01}0

*Conchoraptor gracilis* 1012111111 1101111121 2111111111 1111000111 0?1111111? 1111211111 1111121111 1111111?12 ?011001111 1111121221 110111?201 0010101112 1011110012 1110100021 0001112111 1111100010 101??????? ??110???1? ?11??????? ?????????0 ??????11?0 0?011?0?00 00011?0??0

*Machairasaurus leptonychus* ?????????? ?????????? ?????????? ?????????? ?????????? ?????????? ?????????? ?????????? ?????????? ????????2? ?????????? ?????????? ??????0012 ?????????? ?????????? ?????????? ?????????? ?111110??? ?????????? ????????00 ?????????? ?????????? ??????1???

*Nemegtomaia barsboldi* 1112011111 1101111111 2101111111 1?11000101 1011?11111 1?1??11?11 1111121111 1111111212 ?111001111 11?1121221 110{02}11?201 ?????????? ?????????? ????10002? ?00?1????1 1????????? 1011110101 1?12?11??1 1110000000 1000000100 0??0??1??? 1??11?1?1? 0001?000??

*Heyuannia huangi* ?????????? ?????????? ?????????? ???1?????? ?????????? ?????????? ?????????? ????1??212 1??1001??? ?1??????21 ???21-12?? ??1?????12 1?11000002 ?20???01?1 100111?1?? ?????????? ???1?1?101 1112?2101? 1????????? 0?00000100 ?0?0?0101? 00?1?11?0? ??0?00001?

*Ingenia yanshini* ???????1?? ?????????? ???11????? 1?11000111 ?0111111?? ?111??11?1 ??111?1??? 1111111212 ?011001111 111112122? 110011?201 0010101012 1011000012 0200100121 0001112111 1111100010 ?011?10101 1112121011 1110000000 0000000100 ?0000?1?10 0001?1??0? ??0?000010

*Gigantoraptor erlianensis* ?????????? ?????????? ?????????? ?????????? ?????????? ?????????? ?????????? 1121111202 0111001111 12????1?2? ?????????? 0010?0??00 ?00000?11? 1??1?????? ?????1??11 11111000?0 ???0001010 000??????? ???0???000 1001110100 ???0?????? 1?????110? ?112?10???

*Caenagnathasia martinsoni* ?????????? ?????????? ?????????? ?????????? ?????????? ?????????? ?????????? ??21?1?1?? ??12?????? ????????2? ?????????? ?????????? ?????????? ?????????? ?????????? ?????????? ???0001??? 0????????? ???0111111 ?1???????? ?????????? ?????????? ??0???????

*Elmisaurus elegans* ?????????? ?????????? ?????????? ?????????? ?????????? ?????????? ?????????? ?????????? ?????????? ?????????? ?????????? ?????????? ?????????? ?????????? ?????????? ?????1{01}1?? ?????????? ????????0? ?????????? ?????????? ????1????? ?????????? ?????????1

*Leptorhynchos gaddisi* ?????????? ?????????? ?????????? ?????????? ?????????? ?????????? ?????????? ??21?1?{12}?? ??1{12}?????? ????????2? ?????????? ?????????? ?????????? ?????????? ?????????? ?????101?? ????0????? ?????????? ???0111121 ?1???????? ?????????? ?????????? ?????????1

*Chirostenotes pergracilis* ?????????? ?????????? ?????????? ?????????? ?????????? ?????????? ?????????? ?????????? ?????????? ?????????? ???????111 ?????????? 10?????0?? ??1??11??{12} 1101???11? ?1?1100110 ?????????? ??00010100 0????????? ????????12 ?1111????? ?????????? ??????1000

*Caenagnathus collinsi* ?????????? ?????????? ?????????? ?????????? ?????????? ?????????? ?????????? 1121011102 0112001111 111112??2? ?????????? ?????????? ?????????? ?????????? ?????????? ?????????? ???0001010 0????????? ???1101121 01111111?? ?????????? ???1??010? ?201??????

*Anzu wyliei* ?1??101100 ?1100????? ?????????? ???0?000?? ????1011?? 100?1??1?? 0?011????? 1121011?02 0112001111 111?121221 110211?1?1 0010?02?02 100010???? 1110???1?2 ?1?1102111 11111????? 1?00001010 0000??01?0 ??0?101121 11011111?? 1111?11010 1101?00101 021201110?

*Hagryphus giganteus* ?????????? ?????????? ?????????? ?????????? ?????????? ?????????? ?????????? ?????????? ?????????? ?????????? ?????????? ?????????? ??????0010 ?????????? ?????????? ?????????? ?????????? ?000010??? ?????????? ?????????2 ?????????? ?????????? ??????1???

*Elmisaurus rarus* ?????????? ?????????? ?????????? ?????????? ?????????? ?????????? ?????????? ?????????? ?????????? ?????????? ?????????? ?????????? ??????001? ?????????? ?????????? ?????101?? ?????????? ?000010?0? ?????????? ????????11 ???10????? ?????????? ??????1??1

*Nomingia gobiensis*  ?????????? ?????????? ?????????? ?????????? ?????????? ?????????? ?????????? ?????????? ?????????? ?????????? ??00?1?01? 00101020?? ?????????? ????111011 0111110111 11111????? ?????????? ???????1?0 0????????? ?????????? ?111???0?? ?1???1???? ????1??10?

*Epichirostenotes curriei* ?????????0 1?010????? ?1???????? ?????????? ?????????? 110?1?1??0 ?????????? ?????????? ?????????? ?????????? ???0?1?111 ??1???2??? ?????????? ?????????? ?????021?? ?????????? ?????????? ???????1?? ?????????? ?????????? 111??0?0?? ?1???????? ???????10?

Alberta dentary morph 3 ?????????? ?????????? ?????????? ?????????? ?????????? ?????????? ?????????? ?12111?1?? ??1200???? ????????2? ?????????? ?????????? ?????????? ?????????? ?????????? ?????????? ???0001??? 0????????? ???0111121 ?10??????? ?????????? ??????0??? ??0???????

‘*Caenagnathus*’ *sternbergi* ?????????? ?????????? ?????????? ?????????? ?????????? ?????????? ?????????? 1121111102 0112001111 12?112??2? ?????????? ?????????? ?????????? ?????????? ?????????? ?????????? ???0001010 0????????? ???1111121 11111111?? ?????????? ???1??010? ??0???????

*Macrophalangia canadensis* ?????????? ?????????? ?????????? ?????????? ?????????? ?????????? ?????????? ?????????? ?????????? ?????????? ?????????? ?????????? ?????????? ?????????? ?????????? ?????0011? ?????????? ????????0? ?????????? ?????????? ?????????? ?????????? ?????????0

*Banji long* 11?210111? ??01111021 2111111101 1??11101?? ??0??11?11 11???????? 1111121?01 11??11?212 1??000110? ?1?11?122? 1????????? ?????????? ?????????? ?????????? ?????????? ?????????? 1111100101 1????????? ?110?????? 0?00000??? ?????????? ???00?0?00 0?0???????

*Caudipteryx dongi* ?????????? ?????????? ?????????? ?????????? ?????????? ?????????? ?????????? ?????????? ?????????? ?????????? ?????01?0? ?????????? ??0010?1?2 0100?011?2 011000211? ????100012 ?????????? ??00?00100 0????????? ????????01 ?10?????0? ?1???????? ?????00000

*Ganzhousaurus nankangensis* ?????????? ?????????? ?????????? ?????????? ?????????? ?????????? ?????????? ?1{01}111?21? {12}011001??? ??111???2? ?????????? ?0???0???? ?????????? ?????????? ?????????? ?????0001? ???1?001?1 1???????1? ???????0?0 01?10?0??? ????0????? ??????111? ??0???????

*Jiangxisaurus ganzhouensis* 1????????? ?????????? ????11???? ???1?001?? ??????11?? ?????????? 1??1?????? 111111??10 1?1?0011?1 11????122? ?10??????? ??1??????? 1?1100?0?1 0????0???? ?????????? ?????????? ???1?001?1 0??2?????? ?????????? 0?00000?0? ????????1? 0????????? ?00??00???

*Nankangia jiangxiensis* ?????????? ?????????? ?????????? ?????????? ?????????? ?????????? ?????????? ?11101?2?? ???100???? ????????2? ?????1???1 ??1??0?001 1001?????? ???0111011 1111112110 01111????? ???1101??0 ???????1?0 0??11????? 0?1???1??? ?11??????? 01???11?0? ??0??0?00?

*Ojoraptorsaurus boerei* ?????????? ?????????? ?????????? ?????????? ?????????? ?????????? ?????????? ?????????? ?????????? ?????????? ?????????? ?????????? ?????????? ?????????? ?????02??? ?????????? ?????????? ?????????? ?????????? ?????????? 1????????? ?????????? ??????????

*Shixinggia oblita* ?????????? ?????????? ?????????? ?????????? ?????????? ?????????? ?????????? ?????????? ?????????? ?????????? ?????1?2?1 ??1??????? ?????????? ????110000 000?????1? ???1?????? ?????????? ?????????0 1????????? ?????????? ?????????? ?????????? ??????????

*Similicaudipteryx yixianensis* ?????????? ?????????? ?????????? ?????????? ?????????? ?????????? ?????????? ?????????? ?????????? ?????????? ??0?11?0?1 ?0??1?2??? ????1????? ???0?001?2 1110?????? ???0??0?12 ?????????? ?????????0 0????????? ?????????? ????0????? ?????????? ????10????

*Wulatelong gobiensis* 11?2211?11 ?1?1???021 21?111?1?? 1?111001?? 00??11???? ?????????? ?1111????? ?????????? 101????1?? 11????12?? ?????1???1 00????2??? ???????1?? ????1100?2 11111121?? ?????000?0 1????????? ??0??????0 11???????? ????000??? ?1????1?10 ?101?????? ??????0??0

*Yulong mini*  1020101110 1101101111 2101011101 0?01000111 010?111110 110??0???? ????0????? 1111111212 20110011{01}1 111?12122? 100??1???? 001?10??{01}? ?0000101?2 1010?110?1 0??0??0?1? 0??11?0010 10?1100101 1?11000?10 0110?????? 0?0000010? ??????1??? ???1110?01 000??00???

**(5) References cited in Supporting Information**

Averianov, A., and H.-D. Sues. 2012. Correlation of Late Cretaceous continental vertebrate assemblages in Middle and Central Asia. Journal of Stratigraphy 36:462–485.

Balanoff, A. M., and M. A. Norell. 2012. Osteology of *Khaan mckennai* (Oviraptorosauria: Theropoda). Bulletin of the American Museum of Natural History 372:1–77.

Balanoff, A. M., X. Xu, Y. Kobayashi, Y. Matsufune, and M. A. Norell. 2009. Cranial osteology of the theropod dinosaur *Incisivosaurus gauthieri* (Theropoda: Oviraptorosauria). American Museum Novitates 3651:1–35.

Barsbold, R. 1976. A new Late Cretaceous family of small theropods (Oviraptoridae n. fam.) in Mongolia. Doklady Akademii Nauk SSSR 226:221–223.

Barsbold, R. 1981. Toothless dinosaurs of Mongolia. Transactions of the Joint Soviet-Mongolian Palaeontological Expedition 15:28–39.

Barsbold, R. 1983. Carnivorous dinosaurs from the Cretaceous of Mongolia. Transactions of the Joint Soviet-Mongolian Palaeontological Expedition 19:1–117.

Barsbold, R. 1986. Raubdinosaurier Oviraptoren; pp. 210–223 in E. I. Vorobyeva (ed.), Gerpetologicheskie Issledovaniia v Mongol'skoi Narodnoi Respublike. Akademia Nauk SSSR, Institut Evolitsionnoi Morfologii i Ekologii Zhivotnykh Im. A.M. Severtsova, Moscow.

Barsbold, R. 1988. About the bone crest and the horn cover of the carnivorous-*Oviraptor* dinosaurs. Transactions of the Joint Soviet-Mongolian Palaeontological Expedition 34:77–80.

Barsbold, R., and H. Osmólska. 1999. The skull of *Velociraptor* (Theropoda) from the Late Cretaceous of Mongolia. Acta Palaeontologica Polonica 44:189–219.

Barsbold, R., T. Maryańska, and H. Osmólska. 1990. Oviraptorosauria; pp. 249-258 in D. B. Weishampel, P. Dodson, and H. Osmólska (eds.), The Dinosauria. University of California Press, Berkeley.

Barsbold, R., H. Osmólska, M. Watabe, P. J. Currie, and K. Tsogtbaatar. 2000a. A new oviraptorosaur (Dinosauria, Theropoda) from Mongolia: the first dinosaur with a pygostyle. Acta Palaeontologica Polonica 45:97–106.

Barsbold, R., P. J. Currie, N. P. Myhrvold, H. Osmólska, K. Tsogtbaatar, and M. Watabe. 2000b. A pygostyle from a non-avian theropod. Nature 403:155–156.

Bartlett, J. A. 2004. Taphonomy, geology, and paleoecology of the Sandy Site, an exceptional assemblage in the Maastrichtian Hell Creek Formation of South Dakota. M.Sc. thesis, North Carolina State University, Raleigh, 143 pp.

Burton, D., B. W. Greenhalgh, B. B. Britt, B. J. Kowallis, W. S. J. Elliott, and R. Barrick. 2006. New radiometric ages from the Cedar Mountain Formation, Utah and the Cloverly Formation, Wyoming: implications for contained dinosaur faunas. Geological Society of America Abstracts with Programs 38:52.

Chang, S.-C., H. Zhang, P. R. Renne, and Y. Fang. 2009. High-precision ^40^Ar/^39^Ar age for the Jehol Biota. Palaeogeography, Palaeoclimatology, Palaeoecology 280:94–104.

Clark, J. M., M. A. Norell, and L. M. Chiappe. 1999. An oviraptorid skeleton from the Late Cretaceous of Ukhaa Tolgod, Mongolia, preserved in an avianlike brooding position over an oviraptorid nest. American Museum Novitates 3265:1–36.

Clark, J. M., M. A. Norell, and R. Barsbold. 2001. Two new oviraptorids (Theropoda: Oviraptorosauria), Upper Cretaceous Djadokhta Formation, Ukhaa Tolgod, Mongolia. Journal of Vertebrate Paleontology 21:209–213.

Clark, J. M., M. A. Norell, and T. Rowe. 2002. Cranial anatomy of *Citipati osmolskae* (Theropoda, Oviraptorosauria), and a reinterpretation of the holotype of *Oviraptor philoceratops*. American Museum Novitates 3364:1–24.

Clyde, W. C., S. Ting, K. E. Snell, G. J. Bowen, Y. Tong, P. L. Koch, Q. Li, and Y. Wang. 2010. New paleomagnetic and stable-isotope results from the Nanxiong Basin, China: implications for the K/T boundary and the timing of Paleocene mammalian turnover. The Journal of Geology 118:131–143.

Cracraft, J. 1971. Caenagnathiformes: Cretaceous birds convergent in jaw mechanism to dicynodont reptiles Journal of Paleontology 45:805–809.

Currie, P. J. 1989. The first records of *Elmisaurus* (Saurischia, Theropoda) from North America. Canadian Journal of Earth Sciences 26:1319–1324.

Currie, P. J. 1990. Elmisauridae; pp. 245–248 in D. B. Weishampel, P. Dodson, and H. Osmólska (eds.), The Dinosauria. University of California Press, Berkeley.

Currie, P. J. 2000. Theropods from the Cretaceous of Mongolia; pp. 434–455 in M. J. Benton, M. A. Shishkin, D. M. Unwin, and E. N. Kurochkin (eds.), The Age of Dinosaurs in Russia and Mongolia. Cambridge University Press, Cambridge.

Currie, P. J. 2005. Theropoda, including birds; pp. 367–397 in P. J. Currie and E. B. Koppelhus (eds.), Dinosaur Provincial Park: A Spectacular Ancient Ecosystem Revealed. Indiana University Press, Bloomington.

Currie, P. J., and D. A. Russell. 1988. Osteology and relationships of *Chirostenotes pergracilis* (Saurischia, Theropoda) from the Judith River (Oldman) Formation of Alberta, Canada. Canadian Journal of Earth Sciences 25:972–986.

Currie, P. J., S. J. Godfrey, and L. Nessov. 1994. New caenagnathid (Dinosauria: Theropoda) specimens from the Upper Cretaceous of North America and Asia. Canadian Journal of Earth Sciences 30:2255–2272.

D'Emic, M., and B. Britt. 2008. Reevaluation of the phylogenetic affinities and age of a basal titanosauriform (Sauropoda: Dinosauria) from the Early Cretaceous Cloverly Formation of North America. Journal of Vertebrate Paleontology 28:68A.

Dames, W. 1884. Ueber *Archaeopteryx*. Palaeontologische Abhandlungen 3:119–196.

Dames, W. 1897. Über Brustbein, Schulter- und Beckengürtel der *Archaeopteryx*. Sitzungsberichte der Preussichen Akademie der Wissenschaften 1897:818–834.

de Beer, G. 1954. *Archaeopteryx lithographica,* a study based upon the British Museum specimen. British Museum Publication 224:1–68.

Elzanowski, A. 1999. A comparison of the jaw skeleton in theropods and birds, with a description of the palate in the Oviraptoridae. Smithsonian Contributions to Paleobiology 89: 311–323.

Elzanowski, A. 2001. A novel reconstruction of the skull of *Archaeopteryx*. Netherlands Journal of Zoology 51:207–215.

Elzanowski, A. 2002. Archaeopterygidae (Upper Jurassic of Germany); pp. 129–159 in L. M. Chiappe and L. M. Witmer (eds.), Mesozoic Birds: Above the Heads of Dinosaurs. University of California Press, Berkeley.

Elzanowski, A., and P. Wellnhofer. 1996. Cranial morphology of *Archaeopteryx*: evidence from the seventh skeleton. Journal of Vertebrate Paleontology 16:81–94.

Fanti, F., P. J. Currie, and D. Badamgarav. 2012. New specimens of *Nemegtomaia* from the Baruungoyot and Nemegt formations (Late Cretaceous) of Mongolia. PLoS ONE 7:e31330.

Gilmore, C. W. 1924. A new coelurid dinosaur from the Belly River Cretaceous of Alberta. Canada Geological Survey Bulletin 38:1–12.

Greenwald, M. T. 1971. The lower vertebrates of the Hell Creek Formation (Upper Cretaceous), Harding County, South Dakota. M.Sc. thesis, South Dakota School of Mines and Technology, Rapid City, 76 pp.

He, H. Y., X. L. Wang, Z. H. Zhou, F. Jin, F. Wang, L. K. Yang, X. Ding, A. Boven, and R. X. Zhu. 2006. ^40^Ar/^39^Ar dating of Lujiatun Bed (Jehol Group) in Liaoning, northeastern China. Geophysical Research Letters 33:1–4.

He, T., X.-L. Wang, and Z.-H. Zhou. 2008. A new genus and species of caudipterid dinosaur from the Lower Cretaceous Jiufotang Formation of western Liaoning, China. Vertebrata PalAsiatica 46:178–189.

Heilmann, G. 1927. The Origin of Birds. D. Appleton and Company, London, 210 pp.

Ji, Q., P. J. Currie, M. A. Norell, and S.-A. Ji. 1998. Two feathered dinosaurs from northeastern China. Nature 393:753–761.

Jiang, X.-J., Y.-Q. Liu, S.-A. Ji, X.-L. Zhang, L. Xu, S.-H. Jia, J.-C. Lü, C.-X. Yuan, and M. Li. 2011. Dinosaur-bearing strata and K/T boundary in the Luanchuan-Tantou Basin of western Henan Province, China. Science China: Earth Sciences 54:1149–1155.

Johnson, K. R., D. J. Nichols, and J. H. Hartman. 2002. Hell Creek Formation: a 2001 synthesis; pp. 503-510 in J. H. Hartman, K. R. Johnson, and D. J. Nichols (eds.), The Hell Creek Formation and the Cretaceous-Tertiary Boundary in the Northern Great Plains: An Integrated Continental Record of the End of the Cretaceous. Geological Society of America Special Paper 361. Geological Society of America, Boulder.

Kurzanov, S. M. 1981. An unusual theropod from the Upper Cretaceous of Mongolia. Transactions of the Joint Soviet-Mongolian Palaeontological Expedition 15:39–49.

Kurzanov, S. M. 1982. Structural characteristics of the fore limbs of *Avimimus*. Paleontological Journal 1982:108–112.

Kurzanov, S. M. 1983. New data on the pelvic structure of *Avimimus*. Paleontological Journal 1983:110–111.

Kurzanov, S. M. 1985. The skull structure of the dinosaur *Avimimus*. Paleontological Journal 1985:92–99.

Kurzanov, S. M. 1987. Avimimidae and the problem of the origin of birds. Transactions of the Joint Soviet-Mongolian Palaeontological Expedition 31:5–95.

Longrich, N. R., P. J. Currie, and Z.-M. Dong. 2010. A new oviraptorid (Dinosauria: Theropoda) from the Upper Cretaceous of Bayan Mandahu, Inner Mongolia. Palaeontology 53:945–960.

Longrich, N. R., K. Barnes, S. Clark, and L. Millar. 2013. Caenagnathidae from the Upper Campanian Aguja Formation of West Texas, and a revision of the Caenagnathinae. Bulletin of the Peabody Museum of Natural History 54:23–49.

Lü, J. 2002. A new oviraptorosaurid (Theropoda: Oviraptorosauria) from the Late Cretaceous of southern China. Journal of Vertebrate Paleontology 22:877–883.

Lü, J. 2005. Oviraptorid Dinosaurs from Southern China. Geological Publishing House, Beijing, 200 pp.

Lü, J., and B.-K. Zhang. 2005. A new oviraptorid (Theropoda: Oviraptorosauria) from the Upper Cretaceous of the Nanxiong Basin, Guangdong Province of southern China. Acta Palaeontologica Sinica 44:412–422.

Lü, J., D. Huang, and L. Qiu. 2005. The pectoral girdle and the forelimb of *Heyuannia* (Dinosauria: Oviraptorosauria); pp. 256–273 in K. Carpenter (ed.), The Carnivorous Dinosaurs. Indiana University Press, Bloomington.

Lü, J., L. Yi, H. Zhong, and X. Wei. 2013a. A new oviraptorosaur (Dinosauria: Oviraptorosauria) from the Late Cretaceous of southern China and its paleoecological implications. PLoS ONE 8:e80557.

Lü, J., Y. Tomida, Y. Azuma, Z. Dong, and Y.-N. Lee. 2004. New oviraptorid dinosaur (Dinosauria: Oviraptorosauria) from the Nemegt Formation of southwestern Mongolia. Bulletin of the National Science Museum of Tokyo Series C (Geology & Paleontology) 30:95–130.

Lü, J., P. J. Currie, L. Xu, X. Zhang, H. Pu, and S. Jia. 2013b. Chicken-sized oviraptorid dinosaurs from central China and their ontogenetic implications. Naturwissenschaften 100:165–175.

Makovicky, P. J., and H.-D. Sues. 1998. Anatomy and phylogenetic relationships of the theropod dinosaur *Microvenator celer* from the Lower Cretaceous of Montana. American Museum Novitates 3240:1–27.

Makovicky, P. J., D. Li, K.-Q. Gao, M. Lewin, G. M. Erickson, and M. A. Norell. 2009. A giant ornithomimosaur from the Early Cretaceous of China. Proceedings of the Royal Society B: Biological Sciences 277:191–198.

Mayr, G., B. Pohl, S. Hartman, and D. S. Peters. 2007. The tenth skeletal specimen of *Archaeopteryx*. Zoological Journal of the Linnean Society 149:97–116.

Norell, M. A., and P. J. Makovicky. 1999. Important features of the dromaeosaurid skeleton II: information from newly collected specimens of *Velociraptor mongoliensis*. American Museum Novitates 3282:1–45.

Norell, M. A., P. J. Makovicky, and J. A. Clark. 2004. The braincase of *Velociraptor*; pp. 133–143 in P. J. Currie, E. B. Koppelhus, M. A. Shugar, and J. L. Wright (eds.), Feathered Dragons: Studies on the Transition from Dinosaurs to Birds. Indiana University Press, Bloomington.

Novas, F. E. 1994. New information on the systematics and postcranial skeleton of *Herrerasaurus ischigualastensis* (Theropoda: Herrerasauridae) from the Ischigualasto Formation (Upper Triassic) of Argentina. Journal of Vertebrate Paleontology 13:400–423.

Osborn, H. F. 1924. Three new Theropoda, *Protoceratops* zone, central Mongolia. American Museum Novitates 144:1–12.

Osmólska, H. 1976. New light on the skull anatomy and systematic position of *Oviraptor*. Nature 262:683–684.

Osmólska, H. 1981. Coossified tarsometatarsi in theropod dinosaurs and their bearing on the problem of bird origins. Palaeontologica Polonica 42:79–95.

Osmólska, H., P. J. Currie, and R. Barsbold. 2004. Oviraptorosauria; pp. 165–183 in D. B. Weishampel, P. Dodson, and H. Osmólska (eds.), The Dinosauria, Second Edition. University of California Press, Berkeley.

Ostrom, J. H. 1970. Stratigraphy and paleontology of the Cloverly Formation (Lower Cretaceous) of the Bighorn Basin area, Wyoming and Montana. Bulletin of the Peabody Museum of Natural History 35:1–234.

Ostrom, J. H. 1976. *Archaeopteryx* and the origin of birds. Biological Journal of the Linnean Society 8:91–182.

Owen, R. 1863. On the *Archaeopteryx* of von Meyer, with a description of the fossil remains of a long-tailed species, from the lithographic stone of Solenhofen. Philosophical Transactions of the Royal Society of London 153:33–47.

Parks, W. A. 1933. New species of dinosaurs and turtles from the Upper Cretaceous formations of Alberta. University of Toronto Studies, Geological Series 34:1–33.

Pearson, D. A., T. Schaefer, K. R. Johnson, D. J. Nichols, and J. Hunter. 2002. Vertebrate biostratigraphy of the Hell Creek Formation in southwestern North Dakota and northwestern South Dakota; pp. 145–167 in J. H. Hartman, K. R. Johnson, and D. J. Nichols (eds.), The Hell Creek Formation and the Cretaceous-Tertiary Boundary in the Northern Great Plains: An Integrated Continental Record of the End of the Cretaceous. Geological Society of America Special Paper 361. Geological Society of America, Boulder.

Persons, W. S., IV, P. J. Currie, and M. A. Norell. 2013. Oviraptorosaur tail forms and functions. Acta Palaeontologica Polonica.

Reig, O. A. 1963. La presencia de dinosaurios saurisquios en los 'Estratos de Ischigualasto' (Mesotriásico Superior) de Las Provincias de San Juan y La Rioja (República Argentina). Ameghiniana 3:3–20.

Renne, P. R., A. L. Deino, F. J. Hilgen, K. F. Kuiper, D. F. Mark, W. S. I. Mitchell, L. E. Morgan, R. Mundil, and J. Smit. 2013. Time scales of critical events around the Cretaceous–Paleogene boundary. Science 339:684–687.

Roberts, E. M., A. L. Deino, and M. A. Chan. 2005. ^40^Ar/^39^Ar age of the Kaiparowits Formation, southern Utah, and correlation of contemporaneous Campanian strata and vertebrate faunas along the margin of the Western Interior Basin. Cretaceous Research 26:307–318.

Rothschild, B. M., D. H. Tanke, and T. L. Ford. 2001. Theropod stress fractures and tendon avulsions as a clue to activity; pp. 331–336 in D. H. Tanke and K. Carpenter (eds.), Mesozoic Vertebrate Life. Indiana University Press, Bloomington.

Russell, D. A. 1984. A check list of families and genera of North American dinosaurs. Syllogeus 53:1–35.

Russell, D. A., and M. Manabe. 2002. Synopsis of the Hell Creek (uppermost Cretaceous) dinosaur assemblage; pp. 169–176 in J. H. Hartman, K. R. Johnson, and D. J. Nichols (eds.), The Hell Creek Formation and the Cretaceous-Tertiary Boundary in the Northern Great Plains: An Integrated Continental Record of the End of the Cretaceous. Geological Society of America Special Paper 361. Geological Society of America, Boulder.

Sereno, P. C. 1994. The pectoral girdle and forelimb of the basal theropod *Herrerasaurus ischigualastensis*. Journal of Vertebrate Paleontology 13:425–450.

Sereno, P. C., and F. E. Novas. 1992. The complete skull and skeleton of an early dinosaur. Science 258:1137–1140.

Sereno, P. C., and F. E. Novas. 1994. The skull and neck of the basal theropod *Herrerasaurus ischigualastensis*. Journal of Vertebrate Paleontology 13:451–476.

Smith, D. 1992. The type specimen of *Oviraptor philoceratops*, a theropod dinosaur from the Upper Cretaceous of Mongolia. Neues Jahrbuch für Geologie und Paläontologie Abhandlungen 186:365–388.

Sternberg, C. M. 1932. Two new theropod dinosaurs from the Belly River Formation of Alberta. The Canadian Field-Naturalist 46:99–105.

Sternberg, R. M. 1940. A toothless bird from the Cretaceous of Alberta. Journal of Paleontology 14:81–85.

Sues, H.-D. 1977. The skull of *Velociraptor mongoliensis*, a small Cretaceous theropod dinosaur from Mongolia. Paläontologische Zeitschrift 51:173–184.

Sues, H.-D. 1997. On *Chirostenotes*, a Late Cretaceous oviraptorosaur (Dinosauria: Theropoda) from western North America. Journal of Vertebrate Paleontology 17:698–716.

Sullivan, R. M., S. E. Jasinski, and M. P. A. van Tomme. 2011. A new caenagnathid *Ojoraptorsaurus boerei*, n. gen., n. sp. (Dinosauria, Oviraptorosauria), from the Upper Cretaceous Ojo Alamo Formation (Naashoibito Member), San Juan Basin, New Mexico. New Mexico Museum of Natural History and Science, Bulletin 53:418–428.

Tong, H., and J. Mo. 2010. *Jiangxichelys*, a new nanhsiungchelyid turtle from the Late Cretaceous of Ganzhou, Jiangxi Province, China. Geological Magazine 147:981–986.

Varricchio, D. J. 2001. Late Cretaceous oviraptorosaur (Theropoda) dinosaurs from Montana; pp. 42–57 in D. H. Tanke and K. Carpenter (eds.), Mesozoic Vertebrate Life. Indiana University Press, Bloomington.

Vickers-Rich, P., L. M. Chiappe, and S. Kurzanov. 2002. The enigmatic birdlike dinosaur *Avimimus portentosus*; pp. 65–86 in L. M. Chiappe and L. M. Witmer (eds.), Mesozoic Birds: Above the Heads of Dinosaurs. University of California Press, Berkeley.

Wang, B., and Z. Yang. 2007. Late Cretaceous paleomagnetic results from southeastern China, and their geological implication. Earth and Planetary Science Letters 258:315–333.

Wang, S., C. Sun, C. Sullivan, and X. Xu. 2013. A new oviraptorid (Dinosauria: Theropoda) from the Upper Cretaceous of southern China. Zootaxa 3640:242–257.

Watabe, M., S. Suzuki, and K. Tsogtbaatar. 2006. Geological and geographical distribution of bird-like theropod, *Avimimus* in Mongolia. Journal of Vertebrate Paleontology 26:136A–137A.

Wei, X., H. Pu, L. Xu, D. Liu, and J. Lü. 2013. A new oviraptorid dinosaur (Theropoda: Oviraptorosauria) from the Late Cretaceous of Jiangxi Province, southern China. Acta Geologica Sinica (English Edition) 87:899–904.

Wellnhofer, P. 1974. Das funfte skelettexemplar von *Archaeopteryx*. Palaeontographica Abteilung A 147:169–215.

Wellnhofer, P. 1993. Das siebte exemplar von *Archaeopteryx* aus den Solnhofer Schichten. Archaeopteryx 11:1–47.

Wellnhofer, P. 2008. *Archaeopteryx*: Der Urvogel von Solnhofen. Verlag Dr. Friedrich Pfeil, Munich, 256 pp.

Xu, L., Y. Kobayashi, J. Lü, Y.-N. Lee, Y. Liu, K. Tanaka, X. Zhang, S. Jia, and J. Zhang. 2011. A new ornithomimid dinosaur with North American affinities from the Late Cretaceous Qiupa Formation in Henan Province of China. Cretaceous Research 32:213–222.

Xu, X., and F.-L. Han. 2010. A new oviraptorid dinosaur (Theropoda: Oviraptorosauria) from the Upper Cretaceous of China. Vertebrata PalAsiatica 48:11–18.

Xu, X., X. Zheng, and H. You. 2010. Exceptional dinosaur fossils show ontogenetic development of early feathers. Nature 464:1338–1341.

Xu, X., Y.-N. Cheng, X.-L. Wang, and C.-H. Chang. 2002. An unusual oviraptorosaurian dinosaur from China. Nature 419:291–293.

Xu, X., Q. Tan, J. Wang, X. Zhao, and L. Tan. 2007. A gigantic bird-like dinosaur from the Late Cretaceous of China. Nature 447:844–847.

Xu, X., Q. Tan, S. Wang, C. Sullivan, D. W. E. Hone, F. Han, Q. Ma, L. Tan, and X. Dong. 2013. A new oviraptorid from the Upper Cretaceous of Nei Mongol, China, and its stratigraphic implications. Vertebrata PalAsiatica 51:85–101.

Zanno, L. E., and S. D. Sampson. 2005. A new oviraptorosaur (Theropoda, Maniraptora) from the Late Cretaceous (Campanian) of Utah. Journal of Vertebrate Paleontology 25:897–904.

Zhou, Z.-H., and X.-L. Wang. 2000. A new species of *Caudipteryx* from the Yixian Formation of Liaoning, northeast China. Vertebrata PalAsiatica 38:111–127.

Zhou, Z.-H., X.-L. Wang, F.-C. Zhang, and X. Xu. 2000. Important features of *Caudipteryx* – evidence from two nearly complete new specimens. Vertebrata PalAsiatica 38:242–254.
